# Conditional ablation of MAPK7 expression in chondrocytes impairs endochondral bone formation in limbs and adaptation of chondrocytes to hypoxia

**DOI:** 10.1186/s13578-020-00462-8

**Published:** 2020-09-10

**Authors:** Xiaoming Yang, Dongmei Zhong, Wenjie Gao, Zhiheng Liao, Yuyu Chen, Shun Zhang, Hang Zhou, Peiqiang Su, Caixia Xu

**Affiliations:** 1 The Department of Orthopedics, The First Affiliated Hospital, Sun Yat-sen University, 58 Zhongshan Road 2, Guangzhou, 510080 Guangdong People’s Republic of China; 2 Research Center for Translational Medicine, The First Affiliated Hospital, Sun Yat-sen University, 58 Zhongshan Road 2, Guangzhou, 510080 Guangdong People’s Republic of China; 3 Guangdong Provincial Key Laboratory of Orthopedics and Traumatology, Department of Spine Surgery, The First Affiliated Hospital, Guangzhou, 510080 People’s Republic of China; 4 Department of Orthopaedics, Sun Yat-sen Memorial Hospital of Sun Yat-sen University, Guangzhou, China

**Keywords:** MAPK7, Chondrocytes, Growth plate development, Hypoxia, HIF1α

## Abstract

**Background:**

Long bones of limbs are formed through endochondral bone formation, which depends on the coordinated development of growth plates. Our previous studies have demonstrated that dysfunction of mitogen-activated protein kinase 7 (MAPK7) can cause skeletal dysplasia. However, little is known about the role of MAPK7 in the regulation of proliferation and differentiation of chondrocytes during growth plate development.

**Results:**

Ablation of MAPK7 expression in chondrocytes led to growth restriction, short limbs and bone mass loss in postnatal mice. Histological studies revealed that MAPK7 deficiency increased the apoptosis and decreased the proliferation of chondrocytes in the center of the proliferative layer, where the most highly hypoxic chondrocytes are located. Accordingly, hypertrophic differentiation markers were downregulated in the central hypertrophic layer, beneath the site where abnormal apoptosis was observed. Simultaneously, we demonstrated that hypoxic adaptation and hypoxia-induced activation of hypoxia-inducible factor 1 subunit α (HIF1α) were impaired when MAPK7 could not be activated normally in primary chondrocytes. Concomitantly, vascular invasion into epiphyseal cartilage was inhibited when *Mapk7* was deleted.

**Conclusions:**

We demonstrated that MAPK7 is necessary for maintaining proliferation, survival, and differentiation of chondrocytes during postnatal growth plate development, possibly through modulating HIF1α signaling for adaptation to hypoxia. These results indicate that MAPK7 signaling might be a target for treatment of chondrodysplasia.

## Introduction

All long bones of limbs are formed through endochondral bone formation, where the growth plate, which consists of chondrocytes, is gradually replaced by mineralized bone [1]. Chondrocytes within growth plates originate from the chondrogenic differentiation of mesenchymal stem cells (MSCs) within embryonic limb buds during embryonic development [2, 3]. Endochondral bone formation depends on the highly coordinated development of growth plates, where chondrocytes within the growth plate continuously undergo a life cycle of proliferation, hypertrophy, maturation, and apoptosis [4]. During this process, the proliferation and differentiation of chondrocytes are simultaneously coordinated and tightly regulated to form a uniformly arranged growth plate, resulting in proper skeletogenesis [3]. If the successive steps associated with growth plate development are disrupted, a series of chondrodysplastic phenotypes, such as skeletal deformities and abnormal bone mass, may occur [5, 6]. Proliferation and differentiation of growth plate chondrocytes are regulated by multiple signaling molecules, including Indian hedgehog (IHH), parathyroid hormone-related peptide (PTHrP), and fibroblast growth factor (FGF), which can activate single or multiple intracellular signaling pathways to regulate chondrocytes [7].

Furthermore, as the growth plate is a hypoxic tissue without blood vessels, adaptation to hypoxia is an essential event in growth plate development. Hypoxia-inducible factor 1 (HIF1), a heterodimer consisting of HIF1 subunit α (HIF1α) and HIF subunit β (HIF1β), is the chief mediator of hypoxic adaptation in mammalian cells [8]. HIF1α, which is hydroxylated by hydroxylases and degraded rapidly by proteasomes under normoxia, is the hypoxically responsive component of the heterodimer [9]. Under hypoxic conditions, HIF1α is stabilized and binds to the constitutively expressed HIF1β to enhance the expression of genes involved in cell survival and angiogenesis [10]. HIF1α has been shown to be critical for maintaining the survival of hypoxic chondrocytes; HIF1α deficiency in hypoxic chondrocytes can lead to bone deformities and apoptosis of chondrocytes in the center of the growth plate [11, 12].

Mitogen-activated protein kinase 7 (MAPK7), also known as extracellular signal-regulated kinase 5 (ERK5), belongs to the MAPK family [13]. Other members of the MAPK family include MAPK3 (ERK1), MAPK1 (ERK2), c-Jun amino-terminal kinase (JNK), and p38 [14]. Like other members, MAPK7 is involved in a variety of cellular processes, such as proliferation, differentiation, apoptosis, and development [15]. Activation of any MAPK requires dual phosphorylation of a threonine residue and a tyrosine residue in its TXY motif [13]. In some in vitro systems, MAPKs have been found to have similar functions, while several knockout animal models have shown functional specificity among MAPKs [16, 17]. The ERK1/2 signaling pathway has been shown to be involved in normal skeletal development and the regulation of multiple steps of chondrocyte differentiation during endochondral bone formation in several studies with transgenic mice [18, 19]. Our recent studies revealed that rare coding variants of MAPK7 are associated with a predisposition to scoliosis, and deletion of *mapk7* in zebrafish leads to skeletal dysplasia [20]. However, compared with ERK1/2, the role of MAPK7 in endochondral bone formation has been studied little. Previous studies have revealed that MAPK7 negatively regulates chondrogenic differentiation of bone marrow MSCs in vitro [21, 22]. In addition, a recent study from Japan found that conditional ablation of MAPK7 expression in mouse limb bud mesenchyme using *Prx1*-Cre leads to abnormal embryonic skeletogenesis owing to increased chondrogenic differentiation of MSCs within the embryonic limb bud [23]. Evidence from both in vivo and in vitro studies has indicated that MAPK7 plays a key role in the regulation of chondrogenic differentiation of MSCs. However, the specific roles of MAPK7 in differentiated chondrocytes, especially in regulating their proliferation and differentiation during growth plate development, remain unknown.

In the present study, we deleted *Mapk7* in chondrocytes using *Col2a1*-Cre to gain insight into the role of MAPK7 in the regulation of the life cycle of chondrocytes during growth plate development. Our findings indicate that MAPK7 is necessary for the regulation of postnatal growth plate development and adaptation of chondrocytes to hypoxia, which may be associated with its effects on HIF1α signaling under hypoxia.

## Materials and methods

### Transgenic mice and genotyping

*Mapk7* floxed mice which carried the targeted allele with a couple of loxP sites flanking exons 4, 5, 6, and 7 of *Mapk7* gene were generated by Cyagen Biosciences (Suzhou, China), and schematic diagram of loxp sites was shown in Additional file 1: Fig. S1a. The *Col2a1*-Cre mice were a generous gift from Prof. Xiaochun Bai [6]. All the transgenic mice used were the genetic background of C57/BL6. *Mapk7* floxed mice were intercrossed with C*ol2*-Cre mice to generate mice with *Mapk7* conditional knockout (CKO) in chondrocytes. The genotype of *Mapk7* CKO is *Col2a1*-Cre; *Mapk7*^flox/flox^ and other genotypes of the same fetus are as those of control (CON) mice. Comparisons between CON and *Mapk7* CKO mice were always made between littermates. All transgenic mice were bred in the Laboratory Animal Center of Sun Yat-sen University. The animal experiments were approved by the Animal Ethics Committee of the First Affiliated Hospital of Sun Yat-sen University. Genotyping of mice was conducted by PCR using tail genomic DNA; the primers used are listed in Additional file 1: Table S1. The knock-out efficiency was assessed by detecting the expression of MAPK7 protein in growth plate cartilages.

### Primary chondrocyte culture

Chondrocytes were obtained from distal femoral and proximal tibial growth plates of CON or CKO mice at P1. Epiphyseal cartilage tissues were dissected under a stereo light microscope and digested with 0.25% trypsin for 10 min at 37 °C, followed by digestion with 0.1% collagenase type II (Life Technologies, USA) for 12 h at 37 °C. Chondrocytes were seeded in a 6-well plate and cultured at 37 °C with 5% CO_2_. DMEM/F12 with l-glutamine (Gibco, USA) supplemented with 1% penicillin–streptomycin solution (Gibco, USA) and 10% fetal bovine serum (PAN, Germany) was used as the culture medium. Chondrocytes were used within three passages for the hypoxia studies. Once the chondrocytes had grown to approximately 75% confluence, cells were exposed to normoxic (21% O_2_, 5% CO_2_, and 74% N_2_) or hypoxic (1% O_2_, 5% CO_2_, and 94% N_2_) conditions. ITS culture medium was prepared by adding 1% ITS Liquid Media Supplement (100×) (I3146, Sigma, America) and 50 ng/ml bone morphogenetic protein-2 (120-02C, PeproTech, Amarica) into the culture medium.

### Bone marrow mesenchymal stem cells (BMSCs) culture

Isolation and culture of human BMSCs from healthy volunteer donors as described previously [24]. Mouse BMSCs was isolated from tibias bone marrow of 1-month-old *Mapk7*^flox/flox^ mice. Low-glucose DMEM (Gibco, USA) supplemented with 1% penicillin–streptomycin solution (Gibco, USA) and 10% fetal bovine serum (Gibco, USA) was used as the culture medium. The culture medium supplemented with 10 mmol/L β-glycerophosphate and 50 μg/mL ascorbic acid was used as osteogenic medium. For alizarin red staining, cells were washed with PBS and fixed in 4% paraformaldehyde for 5 min, followed by being washed with PBS. Then, the cells were stained with Alizarin Red Solution (Sigma, A5533) for 10 min at room temperature. Alkaline phosphatase (ALP) staining was performed by using BCIP^®^/NBT-Purple Liquid Substrate System for Membranes (Sigma, B3679) as per manufacturer’s instructions.

### CCK-8 assay

Cell proliferation was analyzed using the Cell Counting Kit-8 (CCK8) assay (Dojindo, Kumamoto, Japan) following the manufacturer’s technical manual. Primary chondrocytes were inoculated at a density of 1 × 10^4^ per well into 96-well plates and cultured at 37 °C with 5% CO_2_ until cells attached to the wall; After 0, 1, 2, 3, 4, 5 and 6 d of incubation at 37 °C with 5% CO_2_, 10 μl of CCK-8 solution was added to each well, and the plates were incubated at 37 °C in 5% CO_2_ for 3 h. The absorbance at 450 nm of each sample was measured using a microplate reader. The values of absorbance were converted to the numbers of cells when the growth curve was drawn.

### Bone micro-structure and BMD measurements

The limbs of P60 male mice were dissected and fixed in 4% paraformaldehyde for 48 h at 4 °C and then stored in 70% ethanol for micro-computed tomography (μCT) scanning (µCT 80, SCANCO Medical AG, Switzerland). Quantitative volumetric measurements of trabecular micro-structures were conducted on the distal femoral metaphysis region of 50 micro-tomographic slices (360 μm below the growth plate), focusing on the primary spongiosa. A Siemens Preclinical Imaging System was used to measure the bone micro-structure parameters, including cortical thickness, bone mineral density (BMD), bone volume/tissue volume ratio, trabecular thickness, trabecular number, trabecular separation, and trabecular pattern factor.

### Alcian blue and Alizarin red staining

Skeletal staining of the limbs at P0 was performed with Alcian blue and Alizarin red [25]. After removing the skin, the limbs of newborn mice were fixed in 95% ethanol overnight and then in acetone overnight, both at room temperature. Subsequently, the limbs were submerged in 0.03% Alcian blue solution (Solarbio, Beijing, China). Two days later, the limbs were destained by washing in 70% ethanol twice and then incubated in 95% ethanol overnight, followed by incubation in 1% KOH solution for 12 h. The limbs were then stained in 0.005% Alizarin red solution (Solarbio, Beijing, China) for 12 h and incubated in a glycerol/1% KOH solution (1:1, v/v) until the specimen was clear. The skeletal samples were photographed with a stereomicroscope (Zessi, Axio zoom v16, Germany) in 100% glycerol, and the distal epiphysis width and length of limbs were analyzed by ImageJ software (version 1.8.0).

### Antibodies

The following antibodies were used for western blotting and immunofluorescence: anti-RUNX2 (12556, Cell Signaling Technology, WB: 1:1000; ab192256, Abcam, IF: 1:1000), anti-COL10A1 (ab58632, Abcam, WB: 1:1000, IF: 1:500), anti-MMP13 (ab39012, Abcam, WB: 1:1000, IF: 1:250), anti-SOX9 (ab185966, Abcam, WB: 1:1000, IF: 1:1000), anti-HIF1α (36169, Cell Signaling Technology, WB: 1:1000; NB100-479, Novus, IF: 1:200), anti-MAPK7 (3372, Cell Signaling Technology, WB: 1:1000, IF: 1:200), anti-p-MAPK7 (3371, Cell Signaling Technology, WB: 1:1000, IF: 1:200), anti-PCNA (13110, Cell Signaling Technology, WB: 1:1000), anti-IHH (ab52919, Abcam, WB: 1:1000), anti-COL2A1 (ab34712, Abcam, WB: 1:1000), anti-VEGFA (19003-1-AP, Proteintech, WB: 1:1000), anti-CD31 (102501, BioLegend, IF: 1:200), anti-GAPDH (60004-1-Ig, Proteintech, WB: 1:2000), anti-β-actin (60008-1-Ig, Proteintech, WB: 1:2000), Anti-OSX (ab22552, WB: 1:3000, Abcam, IF: 1:500), anti-OPN (ab8448, Abcam, WB: 1:1000, IF: 1:250), anti-OCN (ab93876, Abcam, WB: 1:1000, IF: 1:250), anti-CTSK (ab19027, Abcam, IF: 1:500), anti-mouse IgG (7076, Cell Signaling Technology, WB: 1:2000), anti-rabbit IgG (7074, Cell Signaling Technology, WB: 1:2000), anti-rabbit IgG Alexa Fluor 555 (4413, Cell Signaling Technology, IF: 1:2000), anti-mouse IgG Alexa Fluor 555 (4409, Cell Signaling Technology, IF: 1:2000) and anti-rabbit IgG Alexa Fluor 488 (4408, Cell Signaling Technology, IF: 1:2000).

### Histological analysis

Limbs collected from P1, P7, P14, and P60 mice were dissected, skinned, and fixed in 4% paraformaldehyde for 36 h at 4 °C and then decalcified with 10% EDTA (pH 7.3) for 6 weeks, followed by embedding in paraffin or optimal cutting temperature compound (OCT; Sakura, USA). Then the embedded limbs were processed for paraffin sectioning at 4 µm thickness or frozen sectioning at 5 µm thickness. Paraffin sections was dewaxed in xylene and rehydrated in graded concentration of alcohol before staining. For Hematoxylin–eosin (HE) staining, the sections were stained with Hematoxylin for 8 min, followed by rinsing with 1% acid alcohol solution (1% hydrochloric acid in 70% ethanol) for 3 s. After washing with current water for 15 min, sections were stained with 0.5% Eosin for 5 min. For Safranin O staining, the sections were stained with 1% safranin O solution for 2 min. For von Kossa staining, the frozen sections from P1 limbs were air dried and washed with phosphate buffer saline (PBS), followed by incubation with 1% silver nitrate solution under 100 W light bulb for 1 h. After being rinsed in three changes of distilled water, sections were incubated with 5% sodium thiosulfate for 5 min. After washing with distilled water, the nuclei were counterstained with 0.1% nuclear fast red solution (Solarbio, Beijing, China) for 5 min. For tartrate-resistant acid phosphatase (TRAP) staining, the TRAP activity was detected in paraffin sections using the TRAP staining kit (SLBT1113, Sigma) following the manufacturer’s technical manual. All slides of HE staining, Safranin O staining and von Kossa staining were dehydrated through graded alcohol and cleared in xylene. Coverslips were placed with neutral resins (Servicebio, Wuhan, China). The slides were photographed with a microscopy (Olympus, BX63, Japan) and quantification of the secondary ossification center (SOC) areas was conducted using ImageJ software (version 1.8.0). Quantification of the TRAP staining such as osteoclast surface/bone surface (Oc.S/BS) and osteoclast number/bone perimeter (Oc.N/BPm) were measured using Image-Pro Plus 6.0 as previously described [26].

### Immunofluorescence staining

After being sectioned at 5 µm thickness and mounted onto Superfrost Plus slides (Thermo Fisher Scientific, Waltham, USA), frozen sections from P1 limbs were air dried and washed three times with PBS. After incubation in PBS containing 0.1% Triton X-100(TBS-T) for 5 min at room temperature, sections were blocked with 5% BSA in PBS at room temperature for 30 min, followed by incubation with primary antibody overnight at 4 °C. After washing three times with PBS, sections were incubated with species-matched Alexa Fluor 555 for 1 h. The nuclei were counterstained with DAPI (Cell Signaling Technology, Danvers, USA) for 5 min. Slides were washed with PBS, coverslips placed with antifade mounting medium (Servicebio, Wuhan, China) and photographed with a fluorescent microscopy (Olympus, BX63, Japan).

### TUNEL assay

Paraffin sections from P1 and P7 limbs were deparaffinized in xylene, rehydrated through graded alcohols and washed with distilled water. TdT-mediated dUTP nick end labeling (TUNEL) staining was then performed according to the manufacturer’s instructions of the one-step TUNEL Apoptosis Assay kit (Beyotime, Shanghai, China). Briefly, sections were incubated with 20 μg/ml proteinase K for 30 min at 37 °C, followed by washing three times with PBS. And then, TUNEL detective mixture was pipetted on the sections followed by incubation at 37 °C for 60 min in darkness. After washing three times with PBS, All nuclei were counterstained with DAPI. Slides were mounted with antifade mounting medium (Servicebio, Wuhan, China) under coverslips and photographed with a fluorescent microscopy (Olympus, BX63, Japan).

### EdU incorporation assay

P1 mice received a single intraperitoneal injection of 10 mg/g body weight 5-ethynyl-2′-deoxyuridine (EdU; Invitrogen, USA) 2 h before sacrifice. In the EdU labeling-chasing assay, P2 mice were received two injections of EdU within 6 h and sacrificed 48 h after the first injection [27]. Tibiae were harvested, fixed in 4% paraformaldehyde overnight, dehydrated and embedded in paraffin. Paraffin sections were deparaffinize and rehydrated. Visualization of EdU was performed in accordance with the manufacturer’s instructions of Click-iT Plus EdU Alexa Fluor 555 (C10638, Invitrogen), and nuclei were counterstained with DAPI. Slides were photographed with a fluorescent microscopy (Olympus, BX63, Japan) and EdU-positive chondrocytes was analyzed using ImageJ software (version 1.8.0).

### Plasmid construction and transfection

Full-length, wild-type cDNAs of human MAPK7 and human MEK5 were cloned by PCR into the pcDNA3 vector plasmid respectively (Obio corporation, Shanghai, China). To generate the dominant-negative form of MAPK7 (DN-MAPK7), the TEY motif of the wild-type form of MAPK7 (WT-MAPK7) was mutated to AEF. The constitutively active form of MEK5 (CA-MEK5) was generated by incorporating S311D and T315D mutations using the QuikChange mutagenesis kit (Stratagene, Santa Clara, California). Primary chondrocytes were seeded in 6-well plates and transiently transfected with plasmids using Lipofectamine 3000 (Invitrogen). Cells were harvested 48 h after transfection and protein lysates were assayed by western blot.

### Western blot analysis

The proteins of cartilage or chondrocytes were extracted with RIPA buffer (Beyotime, Shanghai, China) containing 1% phosphatase and protease inhibitors (Bimake, Shanghai, China), followed by measurement of protein concentrations using a BCA protein assay (Beyotime, Shanghai, China). After boiling denaturation, the protein samples (30 μg/sample) were subjected to 10% SDS-PAGE, and then transferred to nitrocellulose membranes (Pall, New York, USA). After being blocked with 5% skim milk for 1 h at room temperature, the membranes were probed with primary antibody at 4 °C overnight and then washed three times with TBS-T before incubation with secondary antibody for 1 h at room temperature. Color development was performed using an ECL chemiluminescence detection kit (Beyotime, Shanghai, China) and images of protein bands were captured using a gel imager (GE, ImageQuant Las4000mini, Japan). GAPDH or β-actin served as the internal control for normalization.

### Real-time quantitative PCR

Total RNA was extracted from cartilage or cultured cells with RNAiso Plus reagent (Roche, Switzerland). cDNA was synthesized using 500 ng of total RNA and a PrimeScript RT reagent kit with gDNA Eraser (Takara, Japan) following the manufacturer’s instructions. Quantitative PCR was performed to amplify the cDNA on a Bio-Rad CFX96 Real-Time PCR System using TB Green Premix Taq II (Takara, Japan) and specific primers (Additional file 1: Table S2). Relative expression levels were calculated by the 2^−ΔΔCt^ method. GAPDH or ACTB served as the internal control for normalization.

### Luciferase reporter assay

Primary chondrocytes were seeded in 24-well plates and transfected with 0.5 μg of HIF1α-responsive element luciferase reporter vector (HRE-luc, Addgene, #26731) [28] and 0.05 μg of a *Renilla* luciferase reporter vector using Lipofectamine 3000 (Invitrogen). After transfection for 24 h, chondrocytes were cultured under normoxia or hypoxia for 24 h. Luciferase activity was measured using the Dual-Luciferase Reporter Assay System (E1910, Promega, USA) according to the manufacturer’s instructions. Briefly, after being washed with PBS, the cells were lysed with 200 μl of the lysis buffer for each well and centrifuged to get supernatant. 30 μl supernatant was pipetted into each well of a 96-well plate and mixed immediately with firefly luciferase working solution to detect Luciferase activity. After the addition of 100 μl *Renilla* luciferase working solution to each well, the *Renilla* luciferase activity was detected. The value of luciferase activity was analyzed by a luminometer (TECAN, Sunrise, Austria). The value of relative luciferase activity (RLU) was obtained by the ratio of firefly luciferase activity to *Renilla* luciferase activity.

### ATP measurement

Primary chondrocytes were cultured in 6-well plates under normoxic or hypoxic conditions for 48 h. ATP levels in chondrocytes were measured using the ATP Bioluminescence Assay Kit (Roche, Basel, Switzerland) with a luminometer (TECAN, Sunrise, Austria) following the manufacturer’s protocol. ATP levels were calculated according to an ATP standard curve and normalized by the protein concentrations (mol/g protein).

### Statistical analysis

All shown data are from independent experiments that were performed at least in triplicate. Quantitative measurements are presented as mean ± standard deviation (*SD*) and were calculated using SPSS software (version 20.0), where *P* < 0.05 was considered to indicate statistical significance. Comparisons between two different experimental groups were made using a two-tailed independent Student’s *t* test or paired-sample *t* test. Data from four experimental groups were analyzed by one-way ANOVA followed by Dunnett’s post hoc test.

## Results

### Ablation of MAPK7 expression in chondrocytes results in short limbs in postnatal mice

To study the specific role of MAPK7 in endochondral bone ossification, we generated mice with ablation of MAPK7 expression in chondrocytes by intercrossing *Mapk7*^flox/flox^ mice with *Col2a1*-Cre mice. After Cre recombination, exons 4, 5, 6, and 7 of the *Mapk7* gene were deleted (Additional file 1: Fig. S1a). The genotype of *Mapk7* CKO mice is *Col2a1*-Cre; *Mapk7*^flox/flox^ and other genotypes of the same fetus were the same as those of the CON mice (Additional file 1: Fig. S1b). MAPK7 protein levels in growth plate cartilage of *Mapk7* CKO mice were very low compared with MAPK7 protein levels in CON mice, as evidenced by western blot (Additional file 1: Fig. S1c).

**Fig. 1:**
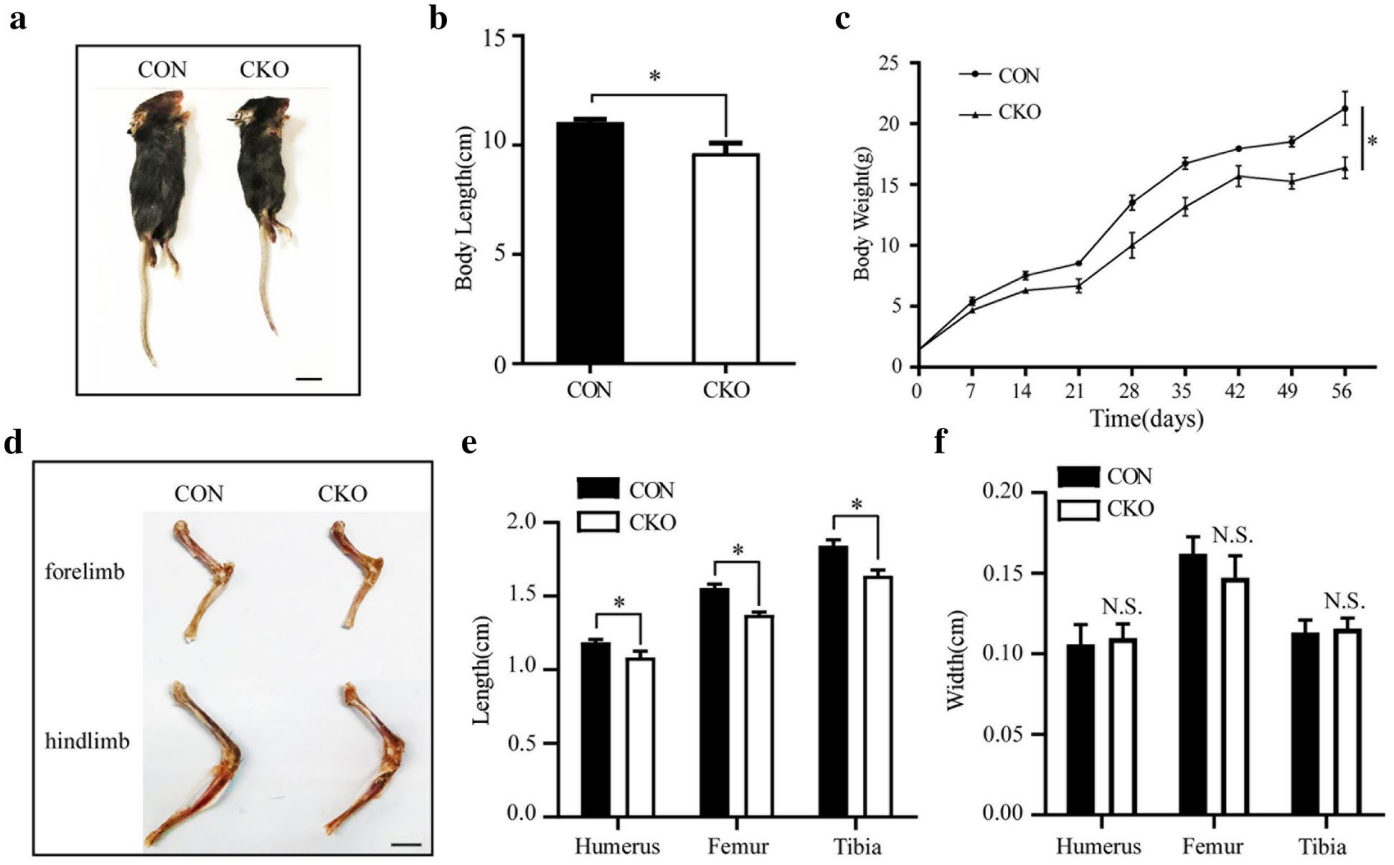
Loss of MAPK7 in chondrocytes caused growth restriction and short limbs in postnatal mice. **a** Phenotype of *Mapk7* CKO and CON littermates at P21. Scale bar, 1 cm. **b** Analysis of body length of *Mapk7* CKO and CON mice at P21 (*n* = 3). *P < 0.05 (Student’s *t* test). Data are presented as mean ± SD. **c** Comparison of body weight between CON and *Mapk7* CKO mice at various time points. Male and female mice were combined (*n* = 4). *P < 0.05 (paired-sample *t* test). Data are presented as mean ± SD. **d** Representative image and (**e, f**) quantification analysis of limb bones from 60-day-old CON and *Mapk7* CKO mice (*n* = 6). Scale bar, 5 mm. *P < 0.05 (Student’s *t* test). N.S., not significant. Data are presented as mean ± SD

*Mapk7* CKO mice were born normally, and no difference in body length at birth (P0) was observed between CON and *Mapk7* CKO mice (Additional file 1: Fig. S2a, b). However, at P21, appearance and body length were significantly different between *Mapk7* CKO and CON mice (Fig. 1a, b). Body weight also showed no difference at P0, but after birth, weight gain was significantly delayed in *Mapk7* CKO mice (Fig. 1c). Furthermore, as shown by Alizarin red/Alcian blue-stained limb bones of newborns, the lengths of the femur, humerus, and tibia were not affected, but *Mapk7* CKO mice had wider upper and lower limbs (Additional file 1: Fig. S2c–e). Nevertheless, in adult P60 mice, the humerus, femur, and tibia of *Mapk7* CKO mice were about 12% shorter than those of CON mice (Fig. 1d, e). There was no statistically significant difference in the width of the limb bones at P60 between these two groups (Fig. 1f). These results indicate that loss of MAPK7 in chondrocytes causes growth restriction and short limbs in postnatal mice.

### Conditional deletion of *Mapk7* in chondrocytes causes loss of bone mass and impairs osteogenesis in limbs

**Fig. 2:**
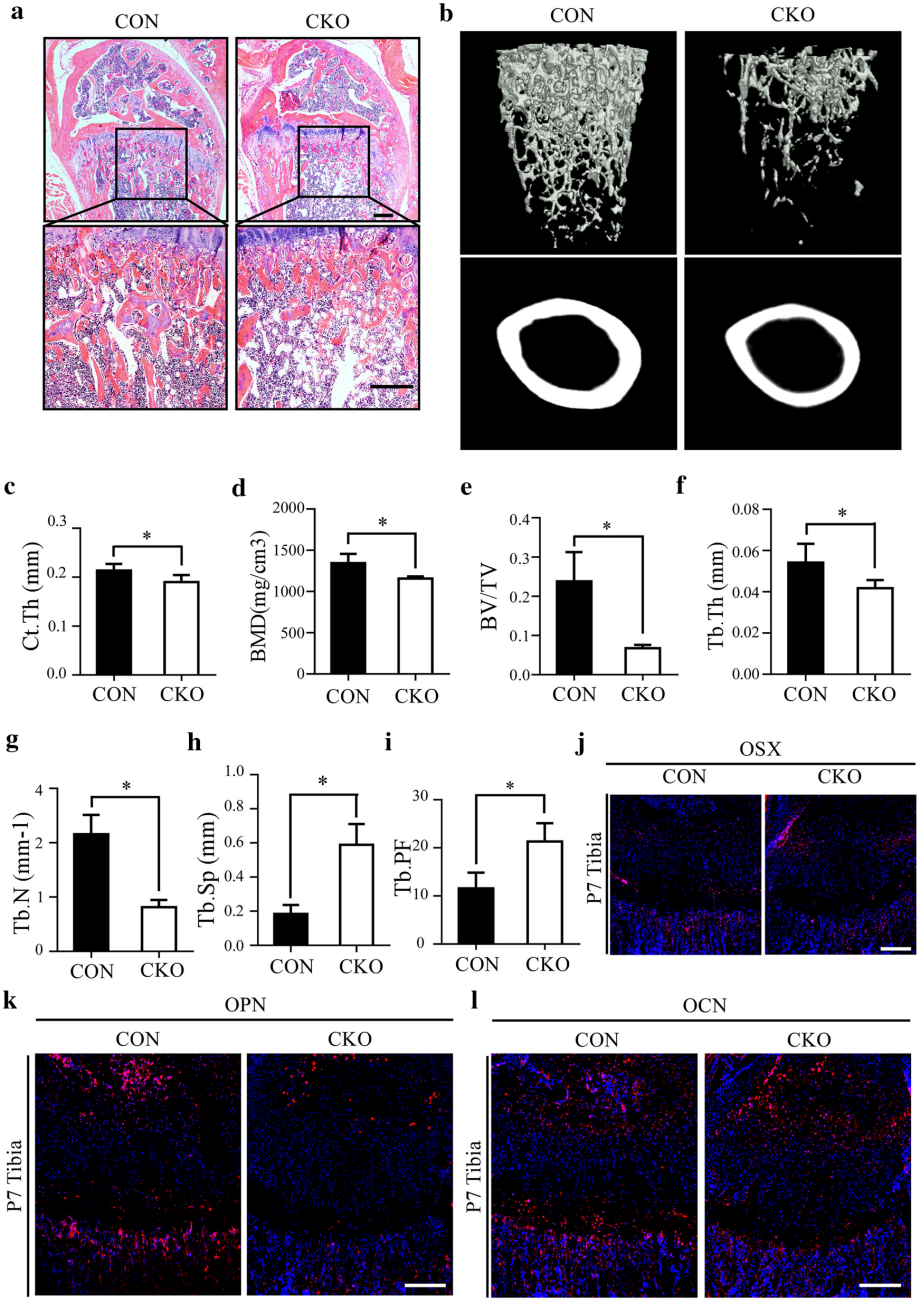
Mice with MAPK7 deficiency in chondrocytes displayed decreased cortical thickness and bone mass loss. **a** HE staining of distal femur sections from P60 mice. Boxed areas are magnified in the images at the bottom. Scale bar, 200 μm. **b** Three-dimensional reconstruction μCT images (*n* = 5) of (upper images) femur trabecular and (lower images) cortical bone in CON and *Mapk7* CKO mice at P60. **c–i** Quantitative μCT analysis of CON and *Mapk7* CKO femurs, including (**c**) cortical thickness, (**d**) bone mineral density (BMD), (**e**) bone volume/tissue volume ratio (BV/TV), (**f**) trabecular thickness, (**g**) trabecular number, (**h**) trabecular separation, and (**i**) the trabecular pattern factor. *P < 0.05 (Student’s *t* test). Data are presented as mean ± SD. **j**–**l** Immunofluorescence staining of (**j**) OSX, (**k**) OPN and (**l**) OCN on representative sections of proximal tibial growth plates from P7 mice

To determine whether conditional deletion of *Mapk7* in chondrocytes affects bone formation, we performed histological and micro-computed tomography (μCT) analysis of the femur at P60. HE staining of distal femurs showed that the amount of trabecular bone mass at the metaphyseal region was significantly reduced in *Mapk7* CKO mice compared with CON mice (Fig. 2a). The trabecular reconstruction of μCT showed that the trabecular bone volume was significantly decreased in *Mapk7* CKO mice (Fig. 2b). The midshaft cortical thickness of *Mapk7* CKO mice was slightly lower than that of CON mice (Fig. 2c). The quantitative analysis of μCT revealed that *Mapk7* CKO mice displayed a reduced bone mineral density (BMD) and bone volume/tissue volume ratio (Fig. 2d, e). Moreover, trabecular number and trabecular thickness were significantly reduced in *Mapk7* CKO mice compared with CON mice (Fig. 2f, g). Conversely, *Mapk7* CKO mice exhibited higher trabecular separation and a higher trabecular pattern factor than CON mice (Fig. 2h, i). Thus, conditional deletion of *Mapk7* in chondrocytes impaired the bone formation of limbs.

To further understand the mechanisms underlying the skeletal defects in the *Mapk7* CKO mice, we examined osteoblast and osteoclast differentiation. In the tibial sections from P7 mice, the expressions of Osterix (OSX), Osteopontin (OPN) and Osteocalcin (OCN), which represent osteogenesis, were reduced at the chondro-osseous junction of *Mapk7* CKO mice (Fig. 2j–l). However, the expression of CTSK, an osteoclast marker, wasn’t increased in the *Mapk7* CKO mice at P1 and P7 (Additional file 1: Fig. S3a, b). We further performed tartrate-resistant acid phosphatase (TRAP) staining to indicate osteoclast activity (Additional file 1: Fig. S3c), and found that there were no difference in osteoclast surface/bone surface (Oc.S/BS) and osteoclast number/bone perimeter (Oc.N/BPm) between 2-month-old *Mapk7* CKO and CON mice (Additional file 1: Fig. S3d, e). These results suggest that *Mapk7* deficiency in chondrocytes impaired osteogenesis, but didn’t affect the erosion of trabecular bone in limbs.

### MAPK7 deficiency reduces survival and proliferation of chondrocytes in the central proliferative layer

**Fig. 3:**
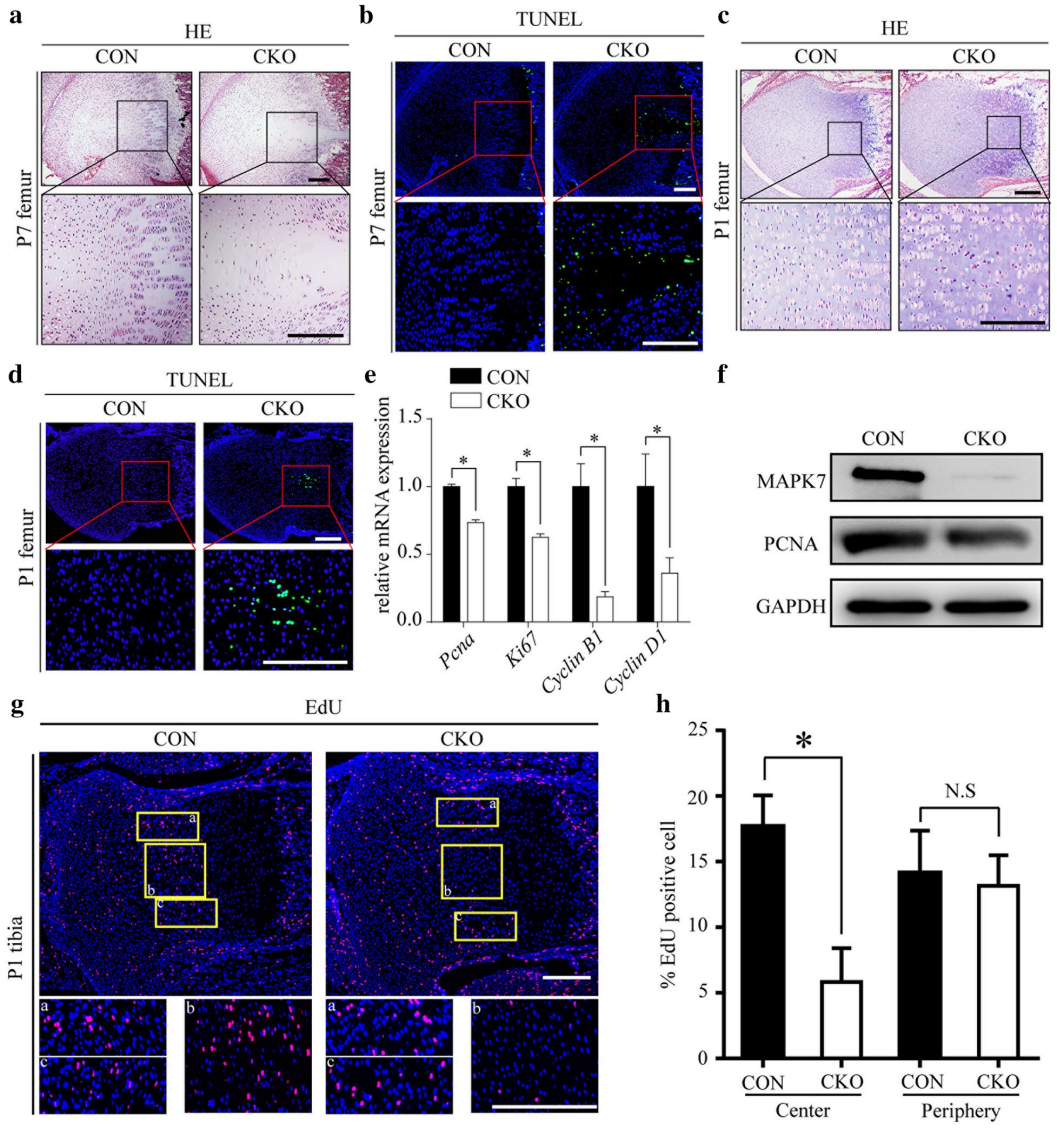
MAPK7 deficiency reduced survival and proliferation of chondrocytes in the central proliferative layer. **a** HE- and (**b**) TUNEL-stained sections of the distal femoral growth plate at P7. Boxed areas in the center of the growth plate are magnified in the images at the bottom. **c** HE- and (**d**) TUNEL-stained sections of the distal femoral growth plates of P1 mice. Boxed areas in the center of the proliferative layer are magnified in the images at the bottom. Scale bar, 200 μm. **e** mRNA levels of *Pcna*, *Ki67*, *Cyclin B1*, and *Cyclin D1* in tibial and femoral growth plates isolated from CON and *Mapk7* CKO mice at P1 were assayed by real-time quantitative PCR (*n* = 3). *P < 0.05 (Student’s *t* test). Data are presented as mean ± SD. **f** Protein levels of MAPK7 and PCNA in tibial and femoral growth plates isolated from CON and *Mapk7* CKO mice at P1 were assayed by western blot. **g** EdU labeling of chondrocytes in tibiae from P1 mice. Boxed areas in the top images, which represent the counted regions in the quantification of EdU-positive cells, are magnified in the images at the bottom. Scale bar, 200 μm. **h** EdU-positive cells were counted in the center and the periphery of the proliferation layer (*n* = 3) *P < 0.05 (Student’s *t* test). N.S., not significant. Data are presented as mean ± SD

To gain insight into how MAPK7 deficiency affects endochondral bone formation, the histological sections of growth plates were studied. After HE staining of femur sections from P7 mice, massive loss of chondrocytes within the center of the proliferating layer could be observed, and *Mapk7* deletion led to hypertrophic layer cleavage (Fig. 3a). To determine whether the reduction in the number of chondrocytes was caused by apoptosis, growth plate sections were TUNEL-stained. The growth plates of *Mapk7* CKO mice showed a marked increase in apoptosis in this hypocellular area compared with CON mice (Fig. 3b). In the P1 growth plates from *Mapk7* CKO mice, the morphology and arrangement of chondrocytes in the center of the proliferative layer were jumbled (Fig. 3c). Consistently, abnormal apoptosis was only observed in the center of the proliferative layer (Fig. 3d). Similar results were obtained for the tibial growth plate (Additional file 1: Fig. S4a, b).

In order to confirm whether MAPK7 deficiency affects chondrocyte proliferation, the expression of some proliferative markers was examined in growth plate cartilage. In the growth plates of *Mapk7* CKO mice, mRNA levels of *Ki67*, *Pcna*, *Cyclin B1*, and *Cyclin D1* were reduced (Fig. 3e), and downregulation of PCNA protein levels also revealed that chondrocyte proliferation was inhibited (Fig. 3f). Moreover, deletion of *Mapk7* inhibited the proliferation in primary cultures of chondrocytes in vitro (Additional file 1: Fig. S4c). To further understand how the proliferation of chondrocytes is altered upon *Mapk7* deletion, EdU was intraperitoneally injected to label proliferative cells in vivo. Consistent with the observed abnormal apoptosis, the proportion of EdU-positive cells was reduced only in the center of the proliferative layer and was not affected in the periphery (Fig. 3g, h). These results indicate that MAPK7 deficiency impaired survival and proliferation of chondrocytes in the central region of the proliferative layer.

### Differentiation of hypertrophic chondrocytes within the central growth plate is impaired in *Mapk7* CKO mice

**Fig. 4:**
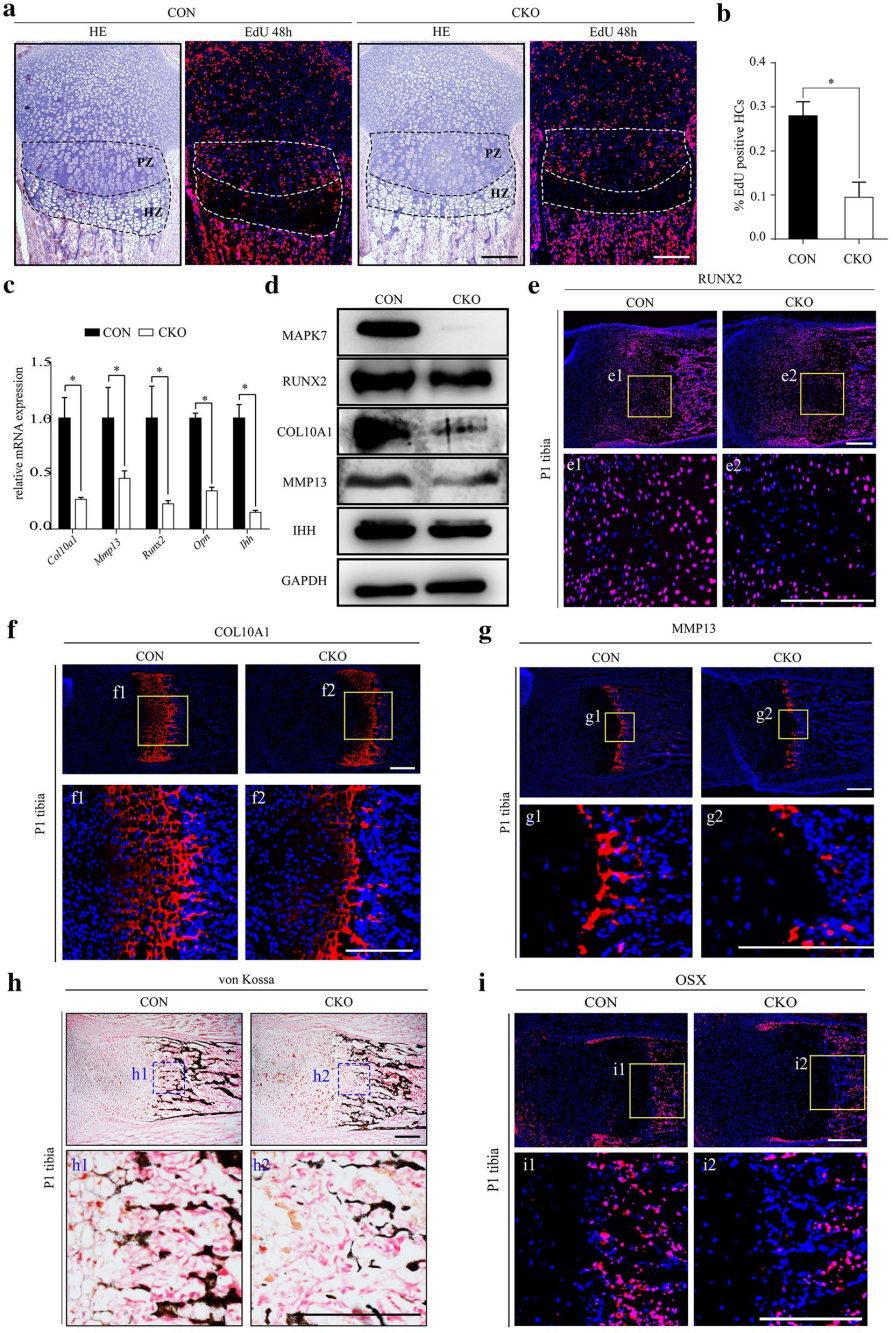
Differentiation of hypertrophic chondrocytes within the central region of growth plates was impaired in *Mapk7* CKO mice. **a** EdU labeling-chasing assay and corresponding HE staining of tibial sections from P4 mice. Dotted lines areas represent the proliferative zone and hypertrophic zone. PZ: proliferative zone; HZ: hypertrophic zone. **b** Quantification of EdU-positive cells in proliferative and hypertrophic zone. The percentage means EdU-labeled in hypertrophic zone relative to total number of EdU-positive chondrocytes in the proliferative and hypertrophic zone. HCs, hypertrophic chondrocytes. (*n* = 5) *P < 0.05 (Student’s *t* test). Data are presented as mean ± SD. **c** Real-time quantitative PCR was performed to measure *Col10a1*, *Mmp13*, *Runx2*, *Opn*, and *Ihh* mRNA expression levels in growth plates from P1 mice (*n* = 3). *P < 0.05 (Student’s *t* test). Data are presented as mean ± SD. **d** Protein levels of MAPK7, COL10A1, MMP13, RUNX2, and IHH in growth plates isolated from CON and *Mapk7* CKO mice at P1 were assayed by western blot. **e**–**g**, **i** Immunofluorescence staining of (**e**) RUNX2, (**f**) COL10A1, (**g**) MMP13, and (**i**) OSX on representative sections of proximal tibial growth plates from P1 mice. Boxed areas are magnified in the images at the bottom. **h** Von Kossa staining of proximal tibial growth plates. Boxed areas are magnified in the images at the bottom. Scale bar, 200 μm

Considering that hypertrophic chondrocytes are derived from cells in the proliferative layer, we also investigated hypertrophic differentiation of growth plate chondrocytes. To determine whether hypertrophic differentiation of proliferative chondrocytes was impaired in *Mapk7* CKO mice, EdU labeling-chasing assay was conducted. We injected EdU into P2 mice twice in 6 h to ensure that the majority of proliferative chondrocytes were labeled. After a chase of 48 h, the number of EdU-labeled hypertrophic chondrocytes that derived from proliferative chondrocytes were significantly reduced in the *Mapk7* CKO growth plates (Fig. 4a). The percentage of EdU-labeled in hypertrophic zone relative to total number of EdU-positive chondrocytes in the proliferative and hypertrophic zone was also decreased (Fig. 4b). This result confirmed that MAPK7 deficiency affects chondrocyte differentiation. mRNA expression levels of *Col10a1*, *Mmp13*, *Runx2*, *Opn*, and *Ihh* were markedly decreased in growth plate cartilage of MAPK7-deficient mice, as evidenced by real-time quantitative PCR (Fig. 4c). Protein expression levels of COL10A1, MMP13, RUNX2, and IHH in cartilage were reduced consistently in MAPK7-deficient mice (Fig. 4d). To confirm whether abnormal chondrocyte differentiation was coupled with impaired survival and proliferation, immunofluorescence staining of differentiation markers and von Kossa staining were performed. When *Mapk7* was deleted, the number of cells expressing RUNX2, a key transcription factor for differentiation, decreased markedly in the central proliferating layer and the hypertrophic layer (Fig. 4e). In addition, in the growth plates of *Mapk7* CKO mice, protein expression levels of COL10A1 and MMP13, which are markers for hypertrophic and terminal hypertrophic chondrocytes, respectively, were decreased in the central region of the hypertrophic layer (Fig. 4f, g). Consistently, von Kossa staining was evidently decreased beneath the centrally located terminal hypertrophic chondrocytes in the absence of MAPK7 (Fig. 4h). In addition to undergoing apoptosis, part of terminal hypertrophic chondrocytes will transdifferentiate into osteoblasts during endochondral bone formation. To understand whether osteoblastic differentiation is affected, the expressions of osteoblast-specific markers at the osteochondral junction were analyzed. Conformably, in the growth plates of *Mapk7* CKO mice at P1, OSX was only down-regulated in the center of chondro-osseous junction compared with wild-type mice (Fig. 4i). However, the expressions of OPN and OCN, two proteins secreted by osteoblasts, were similar in CON and *Mapk7* CKO mice at P1 (Additional file 1: Fig. S5a, b). These results suggested that hypertrophic differentiation of chondrocytes within MAPK7-deficient growth plates was impaired, particularly in the central hypertrophic layer beneath where abnormal apoptosis occurred.

### Activation of MAPK7 in chondrocytes is essential for hypoxic adaptation and enhancement of HIF1α signaling under hypoxia

**Fig. 5:**
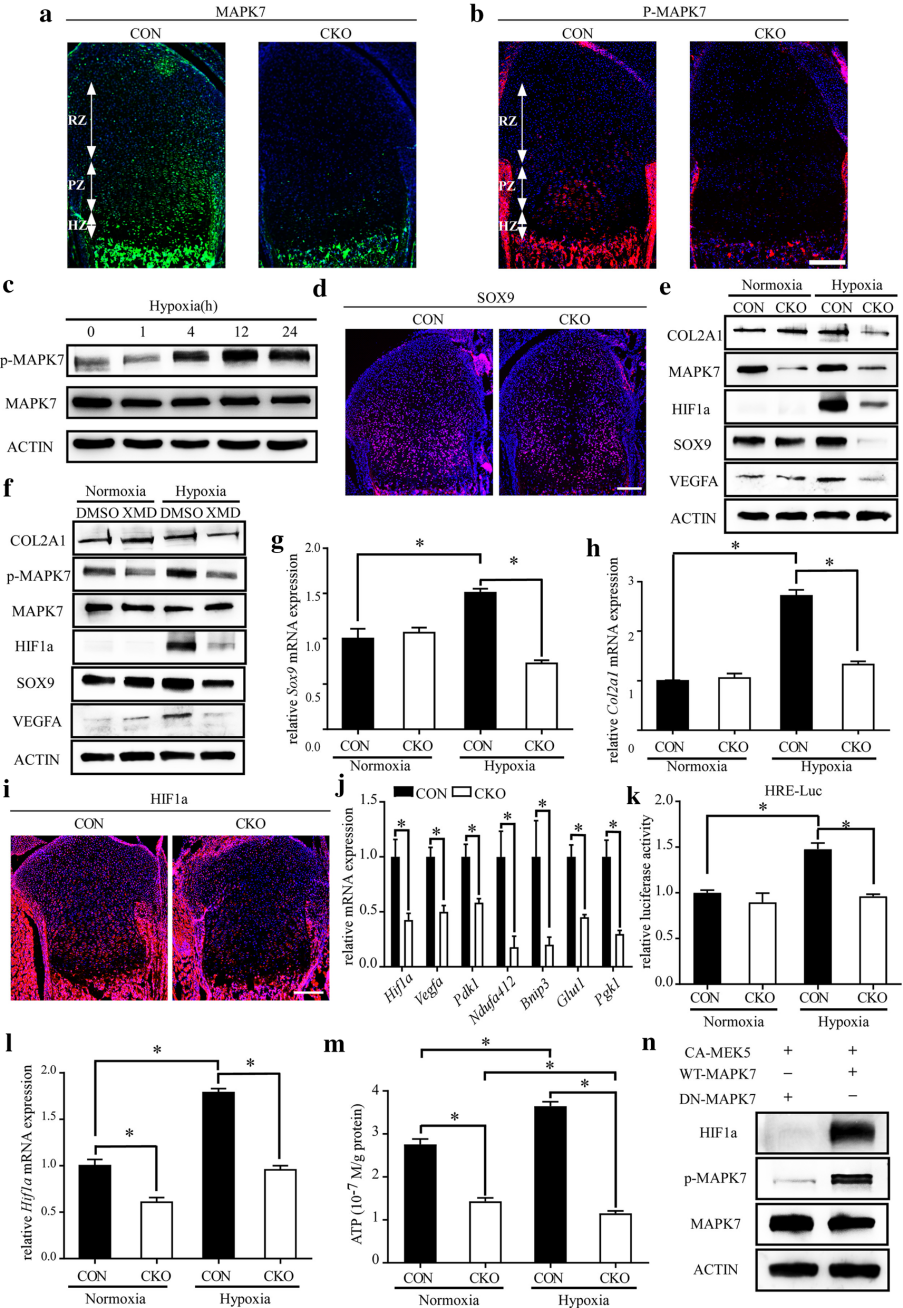
Activation of MAPK7 in chondrocytes is essential for hypoxic adaptation and enhancement of HIF1α signaling under hypoxia. **a**, **b** Immunofluorescence staining of (**a**) MAPK7 (**b**) P-MAPK7 on representative sections of distal femoral growth plates at P1. Scale bar, 200 μm. RZ, resting zone; PZ, proliferative zone; HZ, hypertrophic zone. **c** Western blot analysis of MAPK7 and p- MAPK7 in primary chondrocytes cultured under hypoxic conditions for 0, 1, 4, 12, or 24 h. **d** Immunofluorescence staining of SOX9 on representative sections of distal femoral growth plates at P1. Scale bar, 200 μm. **e** Western blot analysis of SOX9, COL2A1, MAPK7, HIF1α, and VEGFA in CON and *Mapk7* CKO chondrocytes cultured under normoxic or hypoxic conditions for 48 h. **f** Western blot analysis of SOX9, COL2A1, MAPK7, p-MAPK7, HIF1α, and VEGFA protein levels in XMD8-92- or DMSO-treated primary chondrocytes cultured under normoxic or hypoxic conditions. Primary chondrocytes harvested from wild-type mouse knees were cultured in the presence of 5 μM XMD8-92 or an equal amount of DMSO under normoxia for 3 h, followed by normoxia or hypoxia for 48 h. XMD, XMD8-92. **g**, **h** mRNA levels of (**g**) *Sox9* and (**h**) *Col2a1* in CON and *Mapk7* CKO chondrocytes cultured under normoxia or hypoxia were measured by real-time quantitative PCR (n = 3). *P < 0.05 (one-way ANOVA followed by Dunnett’s post hoc test). Data are presented as mean ± SD. **i** Immunofluorescence staining of HIF1α on representative proximal tibial growth plate sections from P1 mice. Scale bar, 200 μm. **j** mRNA levels of *Hif1a* and its known target genes were measured in growth plate cartilage from P1 *Mapk7* CKO mice by real-time quantitative PCR. **k** HIF1α activity in CON and *Mapk7* CKO chondrocytes cultured under normoxia or hypoxia, as evaluated by HIF1α-responsive element luciferase reporter (HRE-luc) assay (*n *= 5). *P < 0.05 (one-way ANOVA followed by Dunnett’s post hoc test). Data are presented as mean ± SD. **l** mRNA level of *hif1a* in CON and *Mapk7* CKO chondrocytes cultured under normoxia or hypoxia were measured by real-time quantitative PCR (n = 3). *P < 0.05 (one-way ANOVA followed by Dunnett’s post hoc test). Data are presented as mean ± SD. **m** Free ATP levels in CON and *Mapk7* CKO chondrocytes cultured under normoxic or hypoxic conditions (*n *= 4). *P < 0.05 (one-way ANOVA followed by Dunnett’s post hoc test). Data are presented as mean ± SD. **n** Primary chondrocytes were transfected with WT-MAPK7 or DN-MAPK7 expression vector in the presence of CA-MEK5 expression vector. Cells were harvested 48 h after transfection and protein levels of HIF1α, MAPK7 and p-MAPK7 were assayed by western blot

The growth plate is a hypoxic tissue, where the most highly hypoxic chondrocytes are located in the center of the proliferative layer and the upper portion of the hypertrophic layer [12]. Abnormalities in chondrocyte proliferation, survival, and differentiation occurring in the hypoxic portion of the growth plate imply that MAPK7 is involved in hypoxic adaptation of chondrocytes. To understand whether MAPK7 and p-MAPK7 are expressed in the most highly hypoxic chondrocytes, we examined the expression patterns of MAPK7 and p-MAPK7 in the sections of growth plate. Immunofluorescence results showed that both MAPK7 and p-MAPK7 were more evident in proliferative and hypertrophic chondrocytes compared with resting chondrocytes (Fig. 5a, b). In addition, MAPK7 was uniformly expressed in the proliferative layer and hypertrophic layer (Fig. 5a), while p-MAPK7 was expressed evidently higher in the center of the proliferative layer and the upper portion of the hypertrophic layer (Fig. 5b). The results implied that the activation of MAPK7 may involve hypoxia. To analyze the dynamic changes in MAPK7 activity in chondrocytes at different oxygen concentrations, primary chondrocytes were exposed to hypoxia for different periods. Total MAPK7 protein levels were not affected under hypoxia, but p-MAPK7 protein levels were significantly increased after 4 h of hypoxia, indicating hypoxic stimulation can increase activation of MAPK7 in chondrocytes (Fig. 5c). We examined the expression of SOX9, which is critical for proliferation, survival, and differentiation of chondrocytes [29, 30], in growth plates. Interestingly, we found that the expression of SOX9 in the growth plates of *Mapk7* CKO mice was only downregulated in the center of the columnar proliferative zone (Fig. 5d). Additionally, mRNA and protein expression levels of SOX9 in MAPK7-deficient chondrocytes were unaffected under normoxic conditions compared with wild-type cultured chondrocytes; however, they were both markedly downregulated under low oxygen conditions (Fig. 5e, f). Similar results were obtained for the expression of collagen type II alpha 1 chain (COL2A1), which is one of target genes regulated by SOX9 (Fig. 5e, h). Subsequently, we blocked MAPK7 signaling with XMD8-92, a specific MAPK7 inhibitor. XMD8-92 treatment only reduced the expression of SOX9 and COL2A1 in chondrocytes under hypoxic conditions (Fig. 5f). Thus, activation of MAPK7 is essential for chondrocytes to maintain the expression of cartilaginous markers under hypoxia, which is important for chondrocytes to adapt to hypoxia [31–33].

HIF1α is vital for chondrocytes to maintain the expression of cartilaginous markers in a hypoxic environment, and its functional activation is important in maintaining the viability of hypoxic chondrocytes located in the central areas of growth plates [12, 34, 35]. We therefore determined whether MAPK7 affects HIF1α signaling in chondrocytes. Immunofluorescence staining showed that HIF1α protein levels were notably reduced in the growth plates of *Mapk7* CKO mice (Fig. 5i). mRNA expression levels of *Hif1a* and its known target genes in cartilage were also downregulated in the absence of MAPK7 (Fig. 5j). Importantly, the protein levels of HIF1α and VEGFA were significantly reduced under hypoxia when *Mapk7* was deleted or its activity was inhibited (Fig. 5e, f). Moreover, HIF1α activity decreased under hypoxia in *Mapk7* CKO chondrocytes (Fig. 5k). The mRNA expression levels of *Hif1a*, *Vegfa*, *Pgk1*, and *Glut1* were increased under hypoxic conditions in the presence of MAPK7, but this increase was not observed in the absence of MAPK7 (Fig. 5l, Additional file 1: S6a–c). Energy generation deficiency was observed in *Hif1a*-null chondrocytes [35], as indicated by a decrease in free ATP levels. We found that free ATP levels in MAPK7-deficient chondrocytes were reduced under both normoxic and hypoxic conditions (Fig. 5m). Moreover, wild-type primary chondrocytes contained slightly higher free ATP levels under hypoxia compared with cells under normoxia, but when *Mapk7* was deleted, a significant reduction in free ATP levels under hypoxia was observed (Fig. 5m). Subsequently, WT-MAPK7 or DN-MAPK7 was co-introduced in the presence of CA-MEK5 in chondrocytes. CA-MEK5 has been reported to directly phosphorylate and activate MAPK7 [36], however, DN-MAPK7 cannot be phosphorylated due to the lack of two phosphorylation sites for MEK5. In the presence of CA-MEK5, compared with DN-MAPK7, overexpression of WT-MAPK7 significantly upregulated the expressions of p-MAPK7 and HIF1α (Fig. 5n). These results indicate that activation of MAPK7 in chondrocytes is essential for upregulation of HIF1α signaling under hypoxia.

### Targeted disruption of *Mapk7* in chondrocytes inhibits vascular invasion into epiphyseal cartilage and delays secondary ossification center formation

**Fig. 6:**
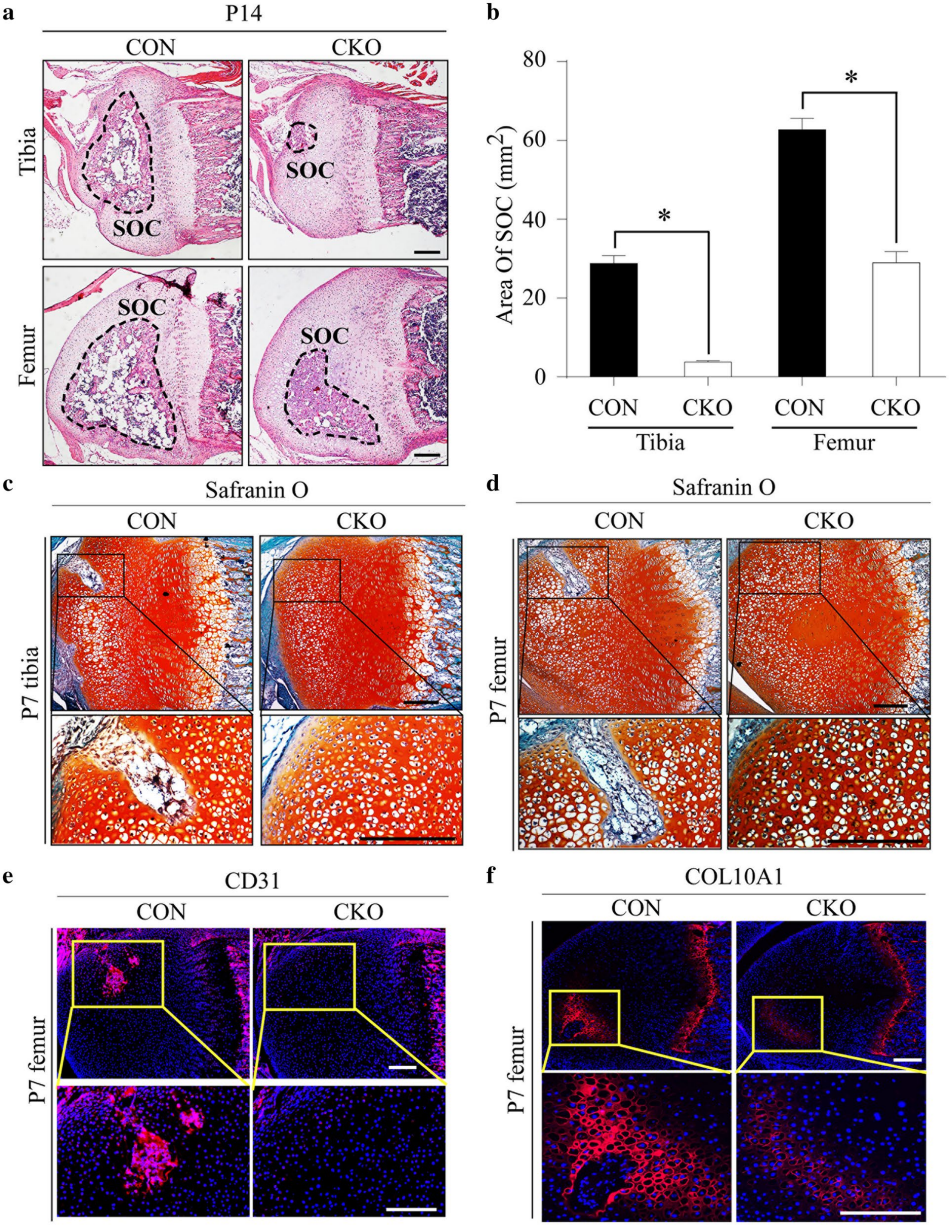
Targeted disruption of *Mapk7* in chondrocytes inhibited vascular invasion into epiphyseal cartilage and delayed secondary ossification center (SOC) formation. **a** HE staining of proximal tibia and distal femur sections from P14 mice. Black dotted lines indicate the contour of the SOC. Scale bar, 200 μm. **b** Quantitative analysis of the areas of the tibial and femoral SOCs from P14 mice (*n* = 4). *P < 0.05 (Student’s *t* test). Data are presented as mean ± SD. **c**, **d** Safranin O staining of proximal tibia and distal femur sections from P7 mice. Boxed areas are magnified in the images at the bottom. Scale bar, 200 μm. **e**, **f** Immunofluorescence staining of (**e**) CD31 and (**f**) COL10A1 on representative sections of distal femurs from P7 mice. Boxed areas are magnified in the images at the bottom. Scale bar, 200 μm

Dysregulation of HIF1α signaling is usually accompanied by abnormalities in vascular invasion into hypoxic tissue [37], while the formation of a secondary ossification center (SOC) begins with vascular invasion into epiphyseal cartilage [1, 4]. To determine whether MAPK7 deficiency can affect the formation of SOCs, sections of epiphyseal cartilage at different times after birth were studied. In the knee sections from P14 mice, in CON mice, SOCs were well formed, in both the tibial and the femoral epiphyseal cartilage; this formation was delayed and SOCs were visually malformed when *Mapk7* was deleted in the growth plates (Fig. 6a). Quantitative analysis showed the area of the tibial and femoral SOCs in *Mapk7* CKO mice decreased to about 10% and 50%, respectively (Fig. 6b). Safranin O staining indicated that blood vessels had invaded the epiphysis of normal tibial and femoral growth plates at P7 in CON mice, which was not observed in the knees of *Mapk7* CKO mice (Fig. 6c, d). The expression of CD31, a key marker of blood vessels, was significantly downregulated in the femoral epiphysis of *Mapk7* CKO mice (Fig. 6e). Moreover, the expression of COL10A1 in epiphyseal cartilage, which should increase significantly after blood vessel invasion, was also significantly inhibited in the absence of MAPK7 (Fig. 6f). These findings suggest that the process of blood vessel invasion into the epiphysis of growth plate cartilage was inhibited and the formation of SOCs was delayed when *Mapk7* was deleted.

## Discussion

Both the embryonic and postnatal growth of long bones in limbs depend on the coordination of endochondral bone formation [1]. In the present study, the lengths of the femur, tibia, and humerus showed no difference between CON and *Mapk7* CKO newborns, but shortness of limbs was observed in adult *Mapk7* CKO mice. The widths of the tibia, femur, and humerus increased in *Mapk7* CKO newborns, but no significant difference was observed between adult CON and *Mapk7* CKO mice. This inconsistency between newborns and adult mice suggests that MAPK7 may play different roles in embryonic and postnatal endochondral bone formation. The role of MAPK7 in endochondral bone formation during embryonic development has been explored by Iezaki et al. [23]. Deletion of *Mapk7* in MSCs using *Prx1*-Cre could result in wider growth plates and long bones at E14.5, E16.5, or E18.5; however, the lengths of long bones at the corresponding development stage were not affected. These results are consistent with our observations of the phenotypes of newborn mice. *Mapk7* was also deleted in chondrocytes within growth plates in *Prx1*-Cre; *Mapk7*^flox/flox^ mice. Combining this previous study with our current findings, we conclude MAPK7 can regulate the width, but not the length, of limb bones during embryonic endochondral bone formation, which is associated with its role in the negative control of the differentiation of MSCs into chondrocytes [23].

To the best of our knowledge, this is the first study of the role of MAPK7 in postnatal endochondral bone formation. Interestingly, shortening of limb bones and growth restriction was observed in postnatal *Mapk7* CKO mice. In addition, the results of μCT revealed the bone mass of femurs was lower in adult mice after *Mapk7* deletion in chondrocytes, though femoral bone mass was not affected in *Prx1*-Cre; *Mapk7*^flox/flox^ embryos at E18.5 [23]. Therefore, we conclude that MAPK7 can regulate the length and bone mass formation of long bones in limbs during postnatal endochondral bone formation. Both increased bone resorption and reduced osteogenesis can lead to loss of bone mass. In the *Mapk7* CKO mice, osteoblast-specific makers were significantly down-regulated at the chondro-osseous junction, but the number and activity of osteoclasts weren’t affected. These results suggest that MAPK7 might be essential for the osteogenic differentiation of terminal hypertrophic chondrocytes. To further confirm whether MAPK7 can regulate the osteogenic differentiation, we induced the osteogenic differentiation of bone marrow mesenchymal stem cells (BMSCs) in vitro (Additional file 1: Fig. S7a–f). Inhibition of MAPK7 activity reduced the osteogenic differentiation of human BMSCs (Additional file 1: Fig. S7a–c). Consistently, the osteogenic differentiation of mouse BMSCs was also obviously impaired when MAPK7 was ablated (Additional file 1: Fig. S7d–f). From these results of in vitro experiments, we found that MAPK7 indeed plays an important role in the regulation of osteogenic differentiation. Performing lineage-tracing experiments to track the fate of chondrocytes in vivo can help determine whether the transdifferentiation of *Mapk7* KO chondrocytes into osteoblasts is impaired.

The most obvious abnormality in the growth plates of *Mapk7* CKO mice was that the area in the center of both the proliferative and the hypertrophic layer was remarkably hypocellular at P7. At P1, the number of chondrocytes in the growth plates of *Mapk7* CKO mice was not decreased significantly, but increased apoptosis in the center of the proliferative and upper hypertrophic layers, as well as decreased proliferation in the center of the proliferative layer, was observed. Hence, the loss of chondrocytes in the center of the proliferative and hypertrophic layers was gradually aggravated from P1 to P7, which was caused by reduced survival and proliferation of chondrocytes within these areas in the absence of MAPK7. Hypertrophic chondrocytes are derived from proliferative chondrocytes, so the decrease in the number of proliferative chondrocytes can also cause the down-regulation of hypertrophic differentiation markers. Therefore, it is necessary to clarify whether the impairment of hypertrophic differentiation is secondary to the reduction of cell proliferation and survival. Our results of EdU labeling-chasing assay confirmed that MAPK7 can indeed regulate the hypertrophic differentiation of proliferative chondrocytes. In addition, reduced differentiation of central chondrocytes may result from the lack of MAPK7 directly or result from impaired hypoxic adaptation caused by MAPK7 deficiency. To get insight into these possibilities, we examined the expression of chondrocyte differentiation markers in CON and *Mapk7* KO chondrocytes under normoxia and hypoxia (Additional file 1: Fig. S8a–c), which indicated MAPK7 deficiency only impaired the expressions of RUNX2 and COL10A1 in hypoxic condition. Eventually, the results of in vitro experiment hinted differentiation retardation caused by MAPK7 deficiency may depend on hypoxia.

The reduced viability of chondrocytes in the central region of growth plates is a unique phenotype caused by decreased HIF1α signaling [11, 12], which has a striking similarity to the abnormalities observed in our study, suggesting that MAPK7 regulates the hypoxic adaptation of chondrocytes and the expression of HIF1α in growth plates. Indeed, the present study provides convincing evidence that hypoxic adaptation and activation of HIF1α signaling under hypoxia are disturbed in MAPK7-deficient chondrocytes. Rebecca et al. [38] found that activation of MAPK7 in human retinal pigment epithelial cells can promote HIF1α signaling by upregulating the mRNA and protein expression levels of HIF1α, which is consistent with our results. However, two other studies carried out in bovine lung micro-vascular endothelial cells and mouse embryonic fibroblast cells showed that MAPK7 activation can inhibit the expression of VEGFA by suppressing HIF1α signaling [39, 40]. These inconsistencies suggest MAPK7 may be able to regulate HIF1α signaling through different molecular mechanisms; this speculation requires further exploration. When HIF1α expression was abolished, chondrocytes within the peripheral proliferative zone where no dead cells could be observed exhibited accelerated proliferation [12]. However, we found that the expression of HIF1α was decreased in the growth plates of *Mapk7* CKO mice. One possible explanation for this discrepancy is that the enhanced proliferation caused by the lack of HIF1α may be rescued by deletion of *Mapk7*. Downregulated expression levels of two cyclin-dependent kinase inhibitors (P21 and P57) due to HIF1α deficiency could explain the enhanced proliferation [12, 41, 42]. In a variety of cancer cells, MAPK7 has been reported to negatively regulate the expression of P21 [43, 44]. Furthermore, we found that the expression of *p21* and *p57* in growth plate cartilage was not affected when *Mapk7* was deleted (Additional file 1: Fig. S9a, b).

Activation of HIF1α signaling can promote angiogenesis and vascular invasion into hypoxic tissues [37], and vascular invasion into the epiphysis is important for SOC formation during postnatal endochondral bone formation [1, 4]. Yang et al. [45] found that overactivation of HIF1α in growth plates can cause excessive invasion of blood vessels into the epiphyseal cartilage in postnatal mice. However, there is not enough evidence to rule out other causes that may inhibit vascular invasion into epiphyseal cartilage in the absence of MAPK7, which is worthy of further study.

**Fig. 7:**
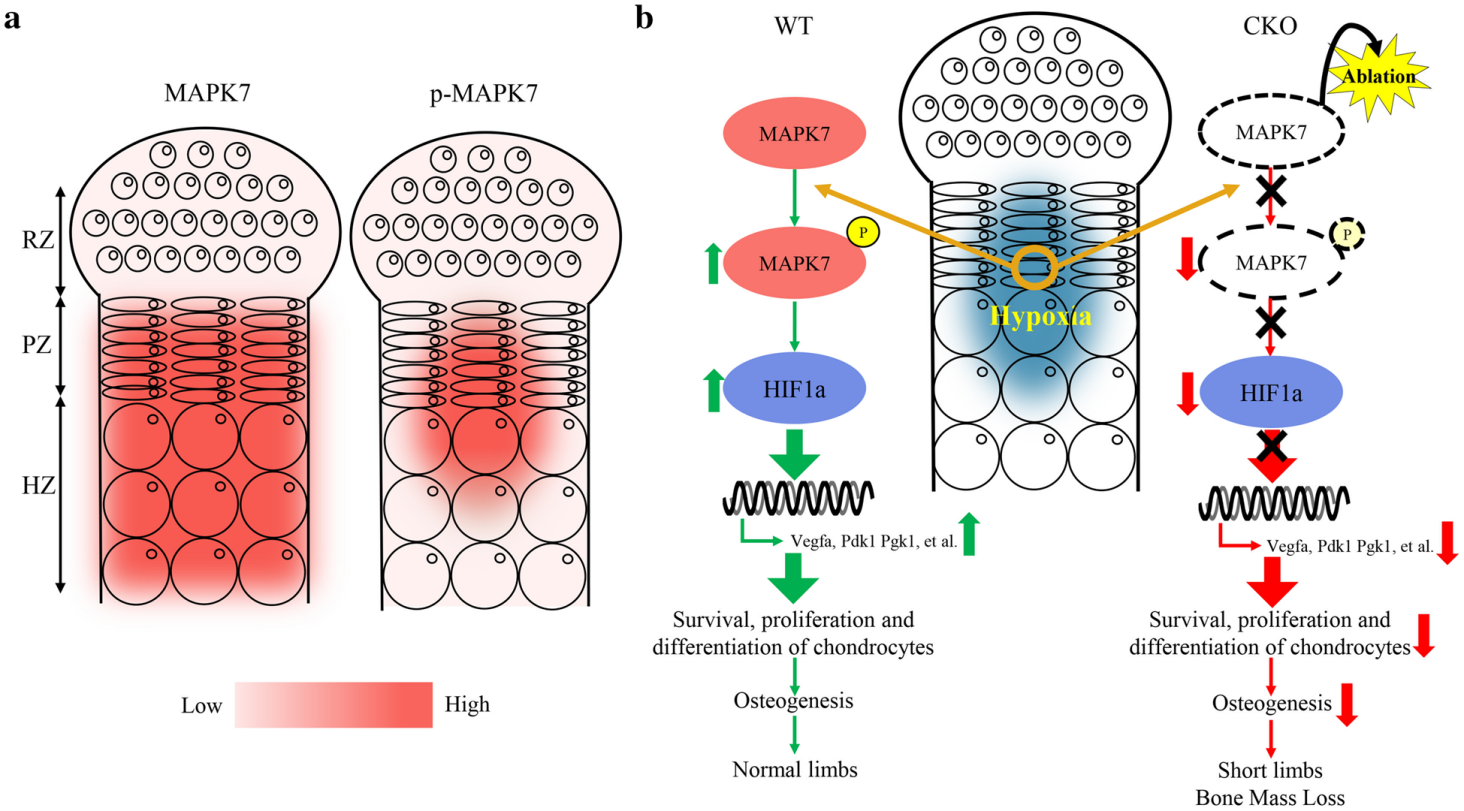
Proposed role of MAPK7 in growth plate development. **a** The expression patterns of MAPK7 and p-MAPK7 in the growth plate. The depth of color represents the expression levels of corresponding markers. RZ, resting zone; PZ, proliferative zone; HZ, hypertrophic zone. **b** A schematic model summarizing the role of MAPK7 in the regulation of growth plate development and modulating HIF1α signaling for hypoxic adaptation

In current study, we first revealed the expression patterns of MAPK7 and p-MAPK7 in the growth plate, which were showed as a thematic diagram in Fig. 7a. MAPK7 was expressed higher in proliferative and hypertrophic chondrocytes of growth plate, compared with resting chondrocytes. During the development of growth plate, proliferative chondrocytes are derived from the differentiation of round chondrocytes in the resting zone, which means this process may be accompanied by an increase in MAPK7 expression. Chondrocytes can be induced to differentiation and maturation in vitro by insulin-transferrin-selenium (ITS) medium, which simulates growth plate development. We isolated epiphyseal chondrocytes and cultured them in ITS medium. After ITS stimulation for 7 days, both MAPK7 and p-MAPK7 were up-regulated (Additional file 1: Fig. S10a, b). Based on in vitro and in vivo results, we speculated that during the process of round chondrocytes in the resting layer differentiating into chondrocytes in the proliferative layer, there may be an unknown molecular mechanism increasing the expression of MAPK7, thus regulating chondrocyte proliferation and hypertrophy. Additionally, compared with the periphery, the higher expression of p-MAPK7 in the center of the proliferative and hypertrophic layer could be explained by the results of in vitro experiments that hypoxia stimulated the activation of MAPK7.

Based on our results, we used a possible small model to summarize our findings (Fig. 7b). During the process of growth plate development, MAPK7 protein expressed in the proliferative and hypertrophic chondrocytes could be activated by hypoxia, which may be essential for the activation of HIF1α signaling to maintain the survival, proliferation and differentiation. When MAPK7 cannot be phosphorylated normally by hypoxia in the *Mapk7* CKO mice, the decreased HIF1A expression and Hif1α signaling activity cause the reduction of the survival, proliferation and differentiation of chondrocytes, which ultimately impairs osteogenesis.

Our study has some limitations that we wish to address. First, in the present study, we have not obtained direct evidence that the abnormal growth plate development in the absence of MAPK7 is caused by an inhibition of HIF1α signaling. Some studies have found that knockout of von Hippel–Lindau tumor suppressor (*Vhl*) and HIF prolyl hydroxylase 2 (*Phd2*), two key genes for HIF1α degradation, can increase HIF1α protein levels [46, 47]. In our future research, we will investigate whether the observed phenotypes can be rescued by deleting *Vhl* and/or *Phd2* in chondrocytes. Second, the molecular mechanisms underlying the regulation of HIF1α signaling by MAPK7 in growth plate chondrocytes remain unknown. Other MAPKs, including ERK1, ERK2, and P38, have been demonstrated to directly phosphorylate HIF1α, enhancing its stability and activity [48, 49]. Therefore, it will be interesting to explore whether MAPK7 can stabilize HIF1α by direct binding and/or phosphorylation.

## Conclusion

In the current study, we explored the role of MAPK7 in the regulation of the development of growth plates during endochondral bone formation of limbs. For the first time, we report that ablation of MAPK7 expression in chondrocytes can result in short limbs, bone mass loss, and dwarfness in postnatal mice. We further showed that the proliferation, survival, and differentiation of the most highly hypoxic chondrocytes located in the central proliferative and hypertrophic layers were significantly impaired in the absence of MAPK7. Furthermore, in vivo and in vitro studies revealed that MAPK7 is essential for hypoxic adaptation and the activation of HIF1α under hypoxia in growth plate chondrocytes. Finally, SOC formation was delayed when *Mapk7* was deleted, which may be associated with the inhibition of HIF1α signaling. Therefore, our data indicate that MAPK7 regulates postnatal endochondral bone formation of limbs, possibly through modulating HIF1α signaling for hypoxic adaptation.

## Supplementary information

**Additional file 1: Fig. S1.** Generation of mice with MAPK7 deficiency in chondrocytes. (a) Schematic diagram of ablation of MAPK7 expression by *Col2a1*-Cre-mediated recombination. The mutant carries the targeted allele with a couple of loxP sites flanking *Mapk7*, and after combining with a mutant carrying the *Col2a1*-Cre recombinase gene, exons 4, 5, 6, and 7 of the *Mapk7* gene were deleted in the chondrocytes. (b) Genotyping of the offspring after breeding transgenic *Col2a1*-Cre and *Mapk7*^flox/flox^ mice. PCR products were detected in homozygous *Mapk7*^flox/flox^ (mutant: 402 bp), wild-type *Mapk7*^+/+^ (wild-type: 285 bp), and *Col2a1*-Cre transgene (mutant: 630 bp) mice. Both 402 bp and 285 bp PCR products were detected in heterozygous mice (*Mapk7*^flox/+^). (c) Western blot analysis of MAPK7 levels in growth plate cartilage isolated from P1 mice. **Fig. S2.** Phenotypes of *Col2a1*; *Mapk7*^flox/flox^ mice at P0. (a, b) Representative lateral view and body length statistics of CON and *Mapk7* CKO mice at P0 (*n* = 3). Scale bar, 1 cm. *P < 0.05 (Student *t* test). N.S., not significant. Data are presented as mean ± *SD*. (c) Alizarin red-/Alcian blue-stained limb bones of CON and *Mapk7* CKO mice at P0. Scale bar, 5 mm. (d, e) Analysis of (d) length and (e) width of femur, humerus, and tibia of CON and *Mapk7* CKO mice at P0 (*n* = 3). **Fig. S3.** Osteoclast-mediated bone-resorbing activity wasn’t elevated in the *Mapk7* CKO mice. (a, b) Immunofluorescence staining of CTSK on representative sections of proximal tibial growth plates from (a) P1 and (b) P7 mice. (c) Tartrate-resistant acid phosphatase (TRAP) staining of tibial primary cancellous bones from P60 mice. (d, e) Quantitative analysis of the TRAP staining. (d) Osteoclast surface/bone surface (Oc.S/BS) and (e) osteoclast number/bone perimeter (Oc.N/BPm) were measured using Image-Pro Plus 6.0 (n = 3). *P < 0.05 (Student’s t test). N.S., not significant. Data are presented as mean ± SD. Scale bar, 200 μm. **Fig. S4.** MAPK7 deficiency impaired survival and proliferation of chondrocytes. (a) HE- and (b) TUNEL-stained sections of proximal tibial growth plates from P1 mice. Boxed areas in the center of the proliferation layer are magnified in the images at the bottom. Scale bar, 200 μm. (c) Cell Counting Kit-8 (CCK-8) assays of CON and *Mapk7* CKO chondrocytes cultured in vitro (*n* = 5). *P < 0.05 (Student’s *t* test). Data are presented as mean ± *SD*. **Fig. S5.** The expression analysis of OPN and OCN at the osteochondral junction of P1 mice. Immunofluorescence staining of (a) OPN and (b) OCN on representative sections of proximal tibial growth plates from P1 mice. Scale bar, 200 μm. **Fig. S6.** MAPK7 deficiency inhibited the increase in mRNA levels of *Vegfa*, *Pgk1*, and *Glut1* induced by hypoxia. mRNA levels of (a) *Vegfa*, (b) *Glut1*, and (c) *Pgk1* in CON and *Mapk7* CKO chondrocytes cultured under normoxia or hypoxia were measured by real-time quantitative PCR (*n* = 3). *P < 0.05 (one-way ANOVA followed by Dunnett’s post hoc test). Data are presented as mean ± *SD*. **Fig.** **S7.** Inhibition of MAPK7 activity and loss of MAPK7 reduced the osteogenic differentiation of bone marrow mesenchymal stem cells. (a–c) Human BMSCs were induced osteoblast differentiation for 14 days in the presence of 5 μM XMD8-92 or an equal amount of DMSO. (a) Osteogenic differentiation of human BMSCs cultured on 6-well plates was determined by alizarin red staining. (b) Real-time quantitative PCR and (c) western blot analyses in the indicated human BMSCs. (n = 3). *P < 0.05 (Student’s t test). Data are presented as mean ± SD. (d-f) Mouse BMSCs harvested from *Mapk7*^*flox/flox*^ mice were infected with Ad-GFP or Ad-Cre for 48 h, followed by being induced osteogenic differentiation for 7 days. Ad, adenovirus. (d) Osteogenic differentiation of mouse BMSCs cultured on 24-well plates was determined by alkaline phosphatase staining. (e) Real-time quantitative PCR and (f) western blot analyses in the indicated mouse BMSCs. (n = 3). *P < 0.05 (Student’s t test). Data are presented as mean ± SD. **Fig. S8.** MAPK7 deficiency inhibited the expressions of RUNX2 and COL10A1 under hypoxia. mRNA levels of (a) *Runx2* and (b) *Col10a1* in CON and *Mapk7* CKO chondrocytes cultured under normoxia or hypoxia were measured by real-time quantitative PCR (n = 3). *P < 0.05 (one-way ANOVA followed by Dunnett’s post hoc test). Data are presented as mean ± SD. (c) Western blot analysis of HIF1α, MAPK7 and RUNX2 in CON and *Mapk7* CKO chondrocytes cultured under normoxic or hypoxic conditions for 48 h. **Fig. S9.** MAPK7 deficiency did not affect the mRNA levels of *p21* and *p57* in growth plate cartilage. Real-time quantitative PCR showed mRNA levels of (a) *p21* and (b) *p57* in growth plate cartilage isolated from CON and *Mapk7* CKO mice at P1 (*n* = 3). *P < 0.05 (Student’s *t* test). N.S., not significant. Data are presented as mean ± SD. **Fig. S10.** Compared with normal medium, MAPK7 and p-MAPK7 were up-regulated when epiphyseal chondrocytes were cultured in ITS medium. (a) Wild-type chondrocytes were cultured in normal or ITS medium for 7 d, and mRNA levels of *Mapk7*, *Col2a1, Col10a1, Runx2, Ihh,* and *Mmp13* were measured by real-time quantitative PCR (n = 3). *P < 0.05 (Student’s *t* test). Data are presented as mean ± SD. (b) The expressions of p-MAPK7, MAPK7, MMP13, and RUNX2 in wild-type chondrocytes cultured in normal or ITS medium on day 7 were analyzed by western blot. ITS, insulin-transferrin-selenium medium. **Table S1.** Primer sequences for genotyping. **Table S2.** Primer sequences for real-time quantitative PCR.

## Data Availability

All data generated or analyzed during this study are included in this article.

## Abbreviations

MAPK7: Mitogen-activated protein kinase 7
p-MAPK7: Phosphorylated MAPK7
CKO: Conditional knockout
COL2A1: Collagen type II alpha 1 chain
HE: Hematoxylin–eosin
μCT: Micro-computed tomography
EdU: 5-Ethynyl-2′-deoxyuridine
TUNEL: TdT-mediated dUTP nick end labeling
IHH: Indian hedgehog
PTHrP: Parathyroid hormone-related peptide
FGF: Fibroblast growth factor
HIF1: Hypoxia-inducible factor 1
HIF1α: Hypoxia-inducible factor 1 subunit α
HIF1β: Hypoxia-inducible factor 1 subunit β
MAPK: Mitogen-activated protein kinase
ERK5: Extracellular signal-regulated kinase 5
JNK: c-Jun amino-terminal kinase
ERK1: Extracellular signal-regulated kinase 1
ERK2: extracellular signal-regulated kinase 2
CON: Control
CCK-8: Cell Counting Kit-8
ANOVA: Analysis of variance
WT-MAPK7: The wild-type form of MAPK7
DN-MAPK7: The dominant-negative form of MAPK7
CA-MEK5: The constitutively active form of MEK5
HRE: HIF1α-responsive element
ATP: Adenosine triphosphate
BMD: Bone mineral density
SOC: Secondary ossification center
TBS-T: Tris-buffered saline with Tween 20
MSC: Mesenchymal stem cell
BMSC: Bone marrow mesenchymal stem cell
VHL: Von Hippel–Lindau tumor suppressor
PHD2: Prolyl hydroxylase 2
RUNX2: Runt-related transcription factor 2
COL10A1: Collagen type X alpha 1 chain
PCNA: Proliferating cell nuclear antigen
MMP13: Matrix metalloproteinase 13
SOX9: SRY-box transcription factor 9
PGK1: Phosphoglycerate kinase 1
VEGFA: Vascular endothelial growth factor A
CD31: Cluster of differentiation 31
Oc.S/BS: Osteoclast surface/bone surface
Oc.N/BPm: Osteoclast number/bone perimeter
TRAP: Tartrate-resistant acid phosphatase
ALP: Alkaline phosphatase
OCN: Osteocalcin
OPN: Osteopontin
OSX: Osterix
CTSK: Cathepsin K
