# Supplementary material for: Conditional ablation of MAPK7 expression in chondrocytes impairs endochondral bone formation in limbs and adaptation of chondrocytes to hypoxia
Source: Cell Biosci. 2020 Sep 10;10:103. doi: 10.1186/s13578-020-00462-8 (PMC7488079; doi:10.1186/s13578-020-00462-8)
Supplement: Supplementary file 1 — Additional file 1: Fig. S1. Generation of mice with MAPK7 deficiency in chondrocytes. (a) Schematic diagram of ablation of MAPK7 expression by Col2a1-Cre-mediated recombination. The mutant carries the targeted allele with a couple of loxP sites flanking Mapk7, and after combining with a mutant carrying the Col2a1-Cre recombinase gene, exons 4, 5, 6, and 7 of the Mapk7 gene were deleted in the chondrocytes. (b) Genotyping of the offspring after breeding transgenic Col2a1-Cre and Mapk7flox/flox mice. PCR products were detected in homozygous Mapk7flox/flox (mutant: 402 bp), wild-type Mapk7+/+ (wild-type: 285 bp), and Col2a1-Cre transgene (mutant: 630 bp) mice. Both 402 bp and 285 bp PCR products were detected in heterozygous mice (Mapk7flox/+). (c) Western blot analysis of MAPK7 levels in growth plate cartilage isolated from P1 mice. Fig. S2. Phenotypes of Col2a1; Mapk7flox/flox mice at P0. (a, b) Representative lateral view and body length statistics of CON and Mapk7 CKO mice at P0 (n = 3). Scale bar, 1 cm. *P < 0.05 (Student t test). N.S., not significant. Data are presented as mean ± SD. (c) Alizarin red-/Alcian blue-stained limb bones of CON and Mapk7 CKO mice at P0. Scale bar, 5 mm. (d, e) Analysis of (d) length and (e) width of femur, humerus, and tibia of CON and Mapk7 CKO mice at P0 (n = 3). Fig. S3. Osteoclast-mediated bone-resorbing activity wasn’t elevated in the Mapk7 CKO mice. (a, b) Immunofluorescence staining of CTSK on representative sections of proximal tibial growth plates from (a) P1 and (b) P7 mice. (c) Tartrate-resistant acid phosphatase (TRAP) staining of tibial primary cancellous bones from P60 mice. (d, e) Quantitative analysis of the TRAP staining. (d) Osteoclast surface/bone surface (Oc.S/BS) and (e) osteoclast number/bone perimeter (Oc.N/BPm) were measured using Image-Pro Plus 6.0 (n = 3). *P < 0.05 (Student’s t test). N.S., not significant. Data are presented as mean ± SD. Scale bar, 200 μm. Fig. S4. MAPK7 deficiency impaired survival and pr [file 13578_2020_462_MOESM1_ESM.docx]

**Supplementary figures**

**
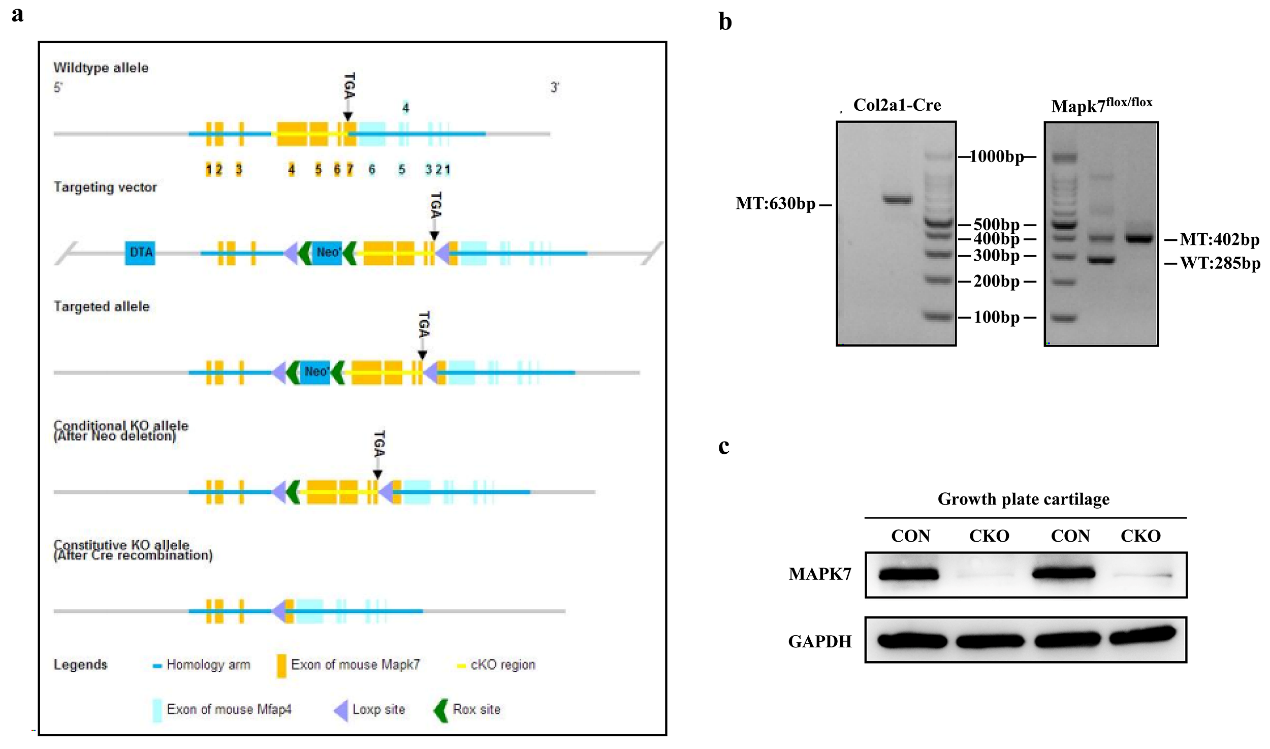
**

**Fig. S1.** Generation of mice with MAPK7 deficiency in chondrocytes. (a) Schematic diagram of ablation of MAPK7 expression by *Col2a1*-Cre-mediated recombination. The mutant carries the targeted allele with a couple of loxP sites flanking *Mapk7*, and after combining with a mutant carrying the *Col2a1*-Cre recombinase gene, exons 4, 5, 6, and 7 of the *Mapk7* gene were deleted in the chondrocytes. (b) Genotyping of the offspring after breeding transgenic *Col2a1*-Cre and *Mapk7*^flox/flox^ mice. PCR products were detected in homozygous *Mapk7*^flox/flox^ (mutant: 402 bp), wild-type *Mapk7*^+/+^ (wild-type: 285 bp), and *Col2a1*-Cre transgene (mutant: 630 bp) mice. Both 402 bp and 285 bp PCR products were detected in heterozygous mice (*Mapk7*^flox/+^). (c) Western blot analysis of MAPK7 levels in growth plate cartilage isolated from P1 mice.


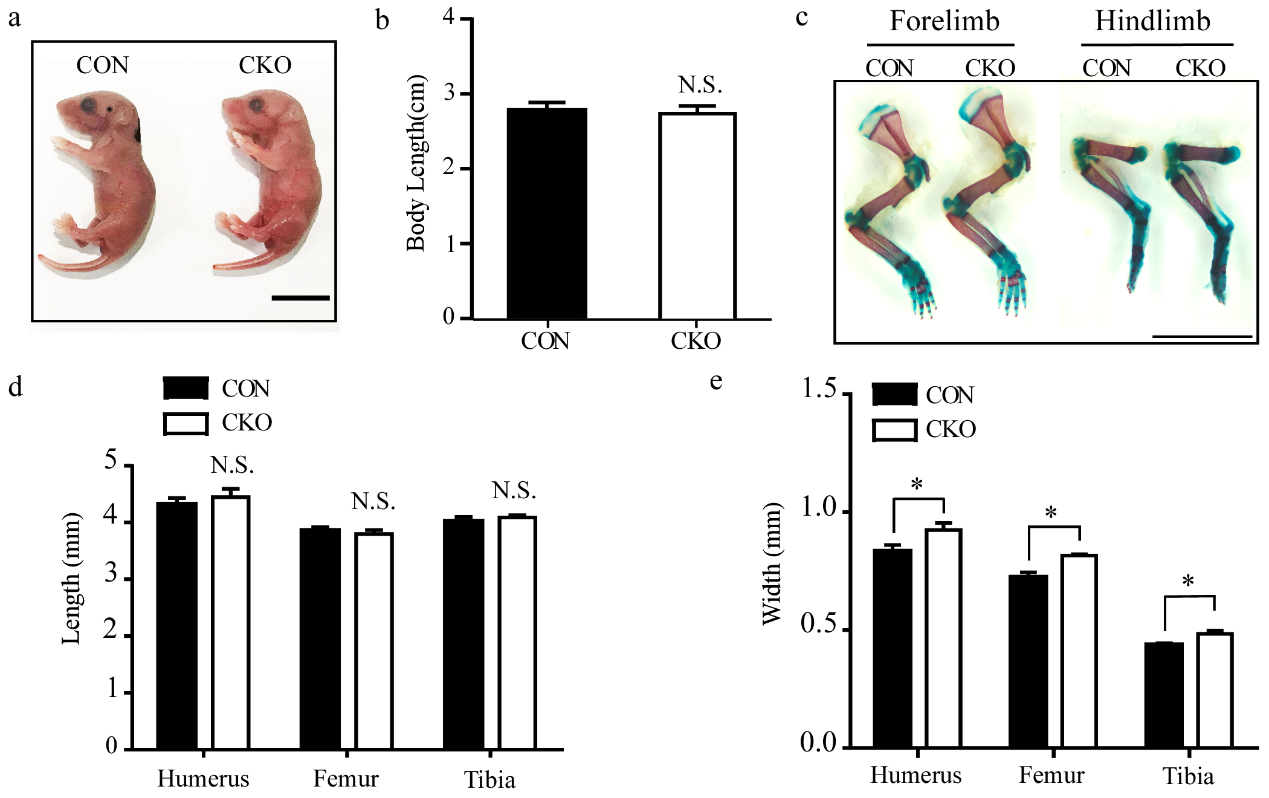


**Fig. S2.** Phenotypes of *Col2a1*; *Mapk7*^flox/flox^ mice at P0. (a, b) Representative lateral view and body length [statistics](D:/Program%20Files%20(x86)/Youdao/Dict/8.8.1.0/resultui/html/index.html#/javascript:;) of CON and *Mapk7* CKO mice at P0 (*n* = 3). Scale bar, 1 cm. **P* < 0.05 (Student *t* test). N.S., not significant. Data are presented as mean ± *SD*. (c) Alizarin red-/Alcian blue-stained limb bones of CON and *Mapk7* CKO mice at P0. Scale bar, 5 mm. (d, e) Analysis of (d) length and (e) width of femur, humerus, and tibia of CON and *Mapk7* CKO mice at P0 (*n* = 3).


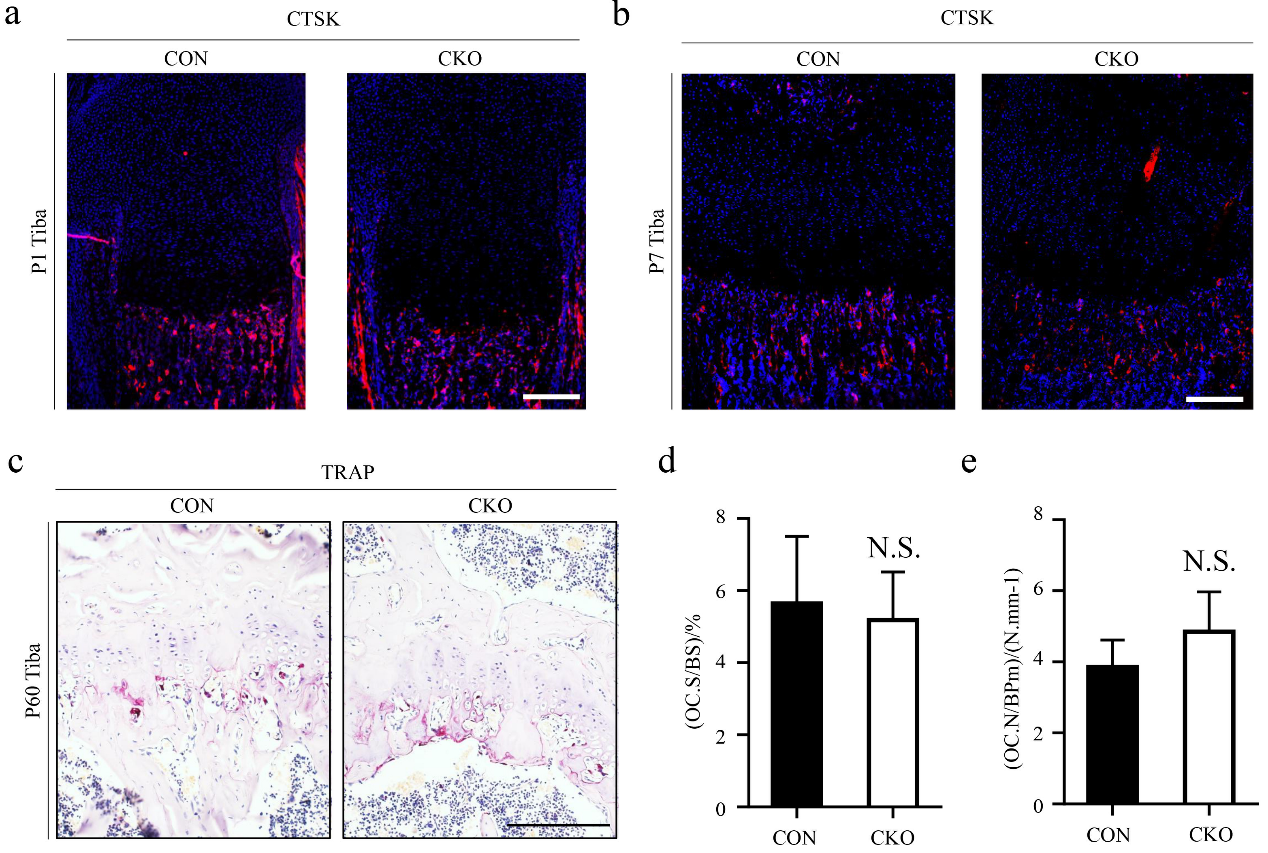


**Fig. S3.** Osteoclast-mediated bone-resorbing activity wasn’t elevated in the *Mapk7* CKO mice. (a, b) Immunofluorescence staining of CTSK on representative sections of proximal tibial growth plates from (a) P1 and (b) P7 mice. (c) Tartrate-resistant acid phosphatase (TRAP) staining of tibial primary cancellous bones from P60 mice. (d, e) Quantitative analysis of the TRAP staining. (d) Osteoclast surface/bone surface (Oc.S/BS) and (e) osteoclast number/bone perimeter (Oc.N/BPm) were measured using Image-Pro Plus 6.0 (n = 3). *P < 0.05 (Student’s t test). N.S., not significant. Data are presented as mean ± SD. Scale bar, 200 μm.


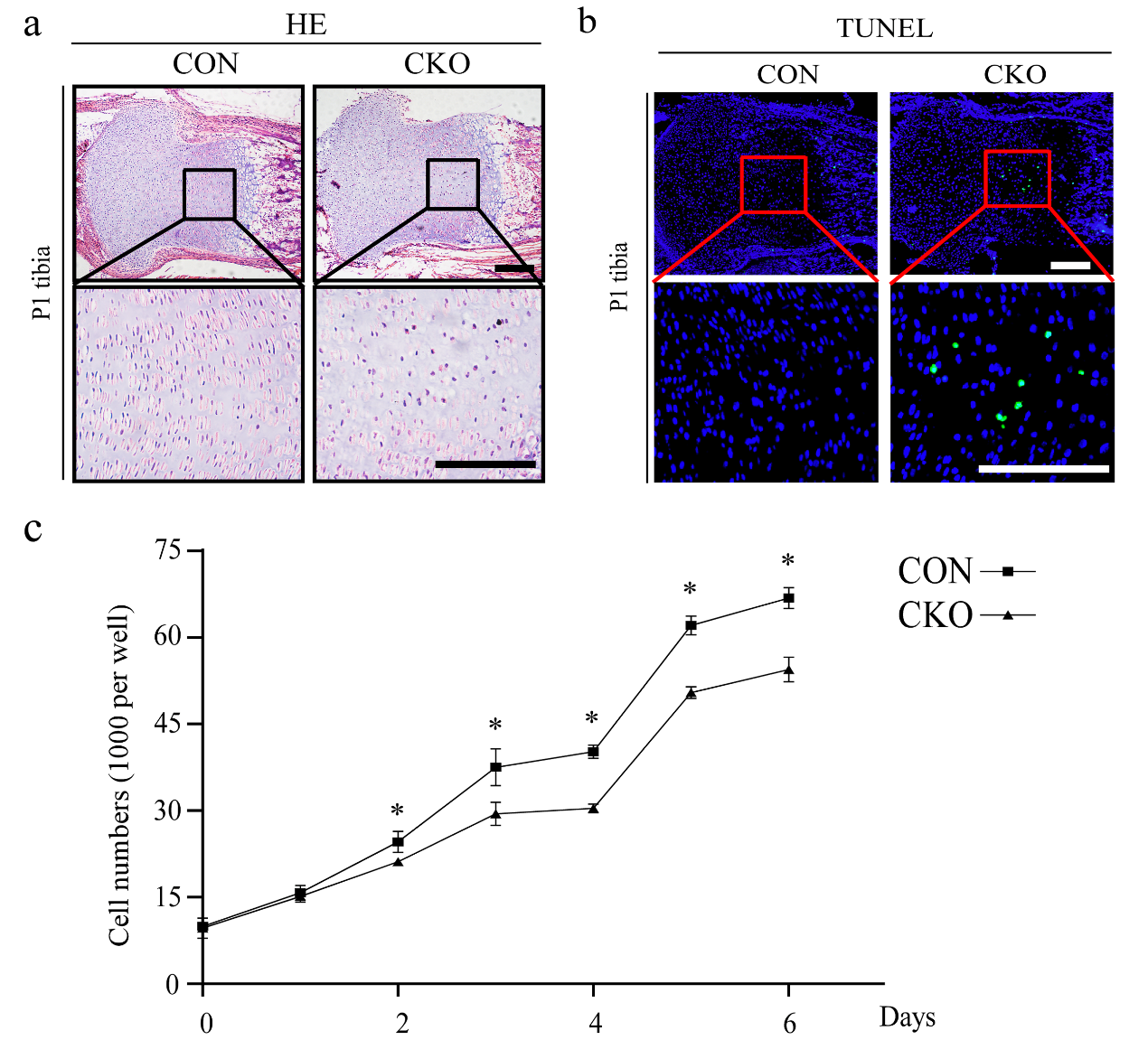


**Fig. S4.** MAPK7 deficiency impaired survival and proliferation of chondrocytes. (a) HE- and (b) TUNEL-stained sections of proximal tibial growth plates from P1 mice. Boxed areas in the center of the proliferation layer are magnified in the images at the bottom. Scale bar, 200 μm. (c) Cell Counting Kit-8 (CCK-8) assays of CON and *Mapk7* CKO chondrocytes cultured in vitro (*n* = 5). **P* < 0.05 (Student’s *t* test). Data are presented as mean ± *SD*.


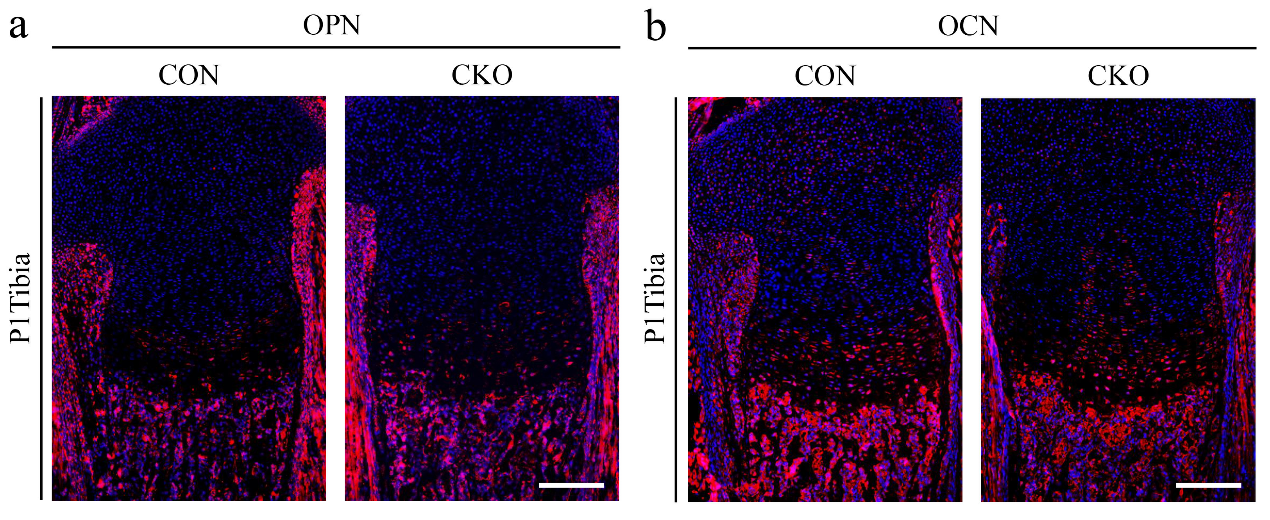


**Fig. S5.** The expression analysis of OPN and OCN at the osteochondral junction of P1 mice. Immunofluorescence staining of (a) OPN and (b) OCN on representative sections of proximal tibial growth plates from P1 mice. Scale bar, 200 μm.


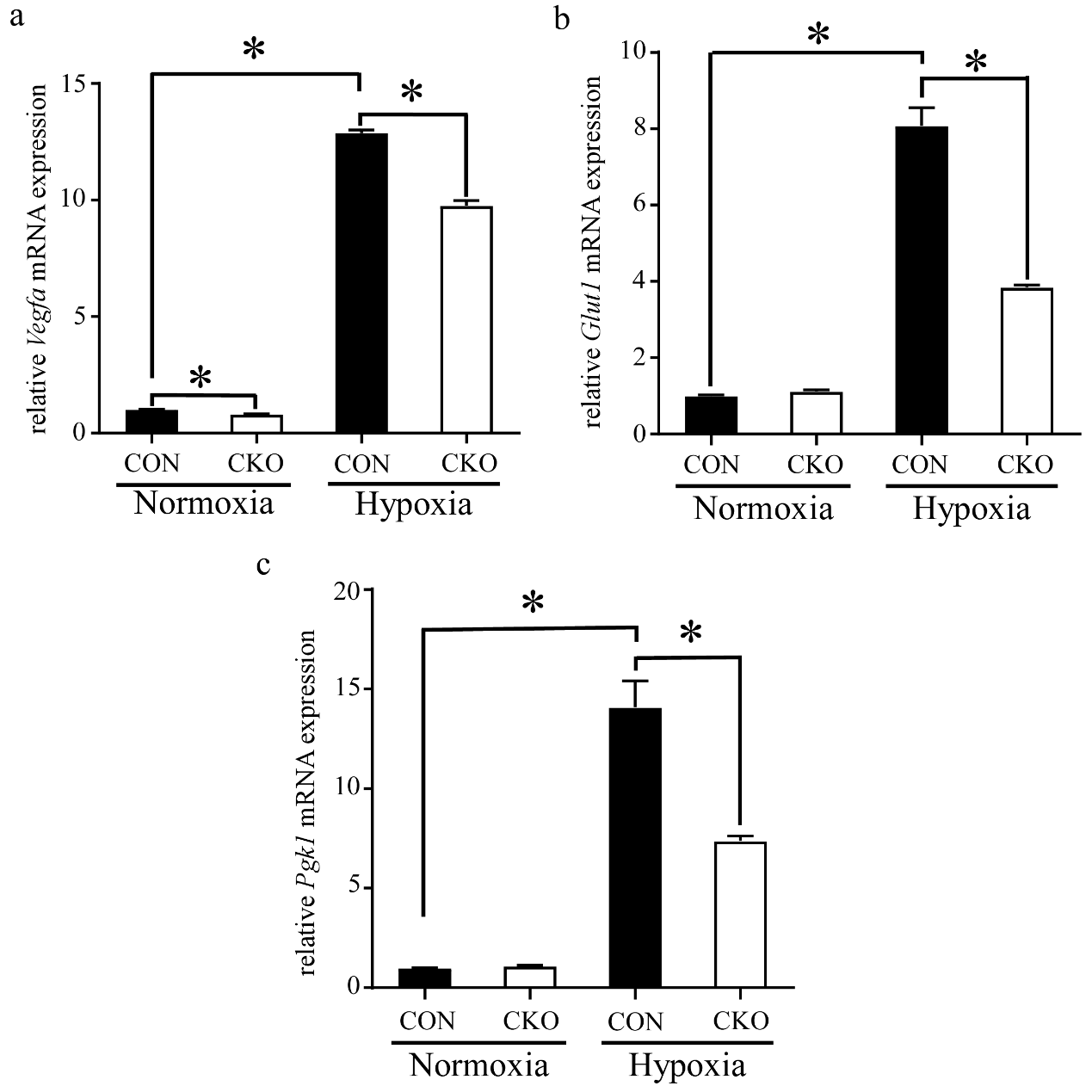


**Fig. S6.** MAPK7 deficiency inhibited the increase in mRNA levels of *Vegfa*, *Pgk1*, and *Glut1* induced by hypoxia. mRNA levels of (a) *Vegfa*, (b) *Glut1*, and (c) *Pgk1* in CON and *Mapk7* CKO chondrocytes cultured under normoxia or hypoxia were measured by real-time quantitative PCR (*n* = 3). **P* < 0.05 (one-way ANOVA followed by Dunnett’s post hoc test). Data are presented as mean ± *SD*.

**
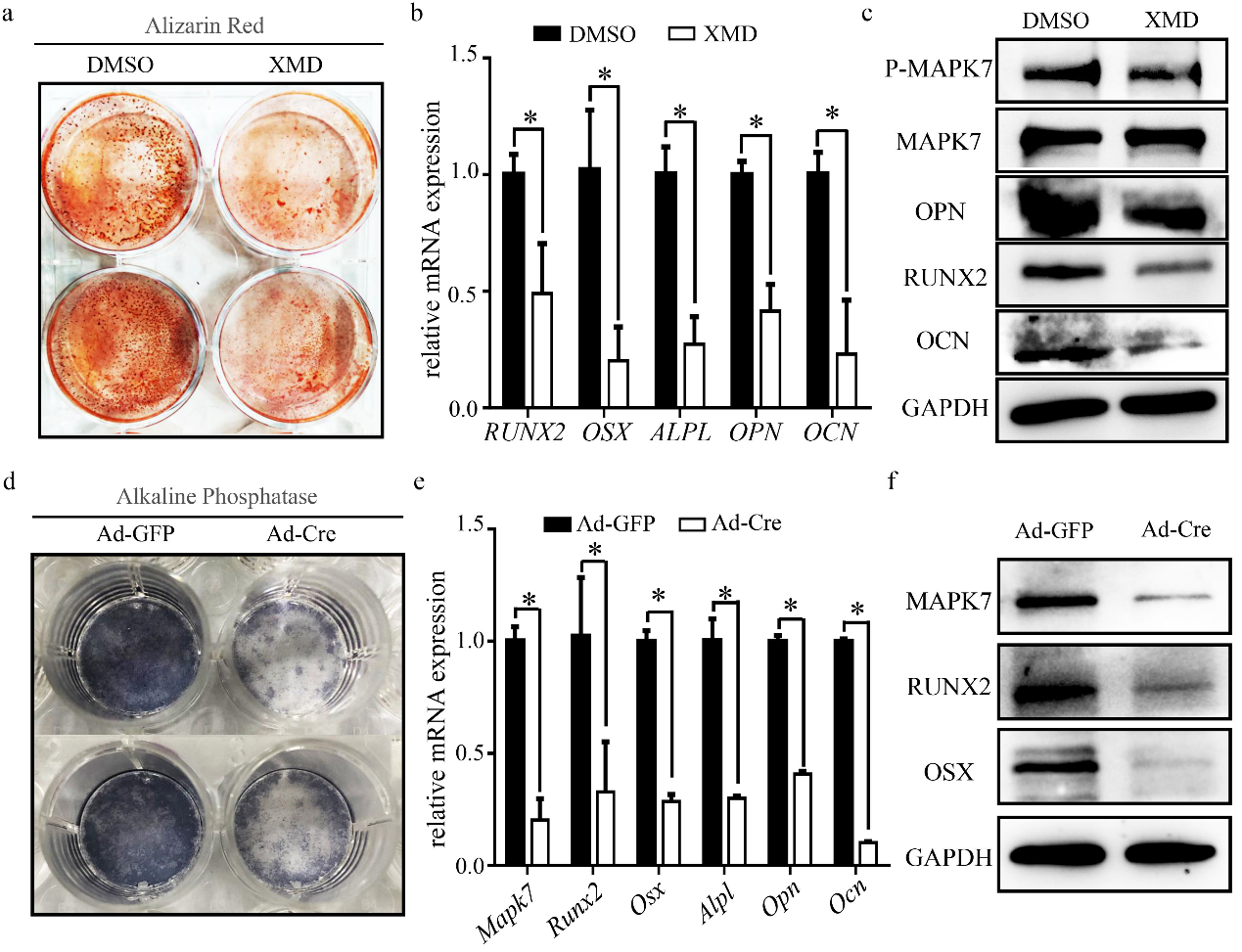
**

**Fig. S7.** Inhibition of MAPK7 activity and loss of MAPK7 reduced the osteogenic differentiation of bone marrow mesenchymal stem cells. (a-c) Human BMSCs were induced osteoblast differentiation for 14 days in the presence of 5 μM XMD8-92 or an equal amount of DMSO. (a) Osteogenic differentiation of human BMSCs cultured on 6-well plates was determined by alizarin red staining. (b) Real-time quantitative PCR and (c) western blot analyses in the indicated human BMSCs. (n = 3). *P < 0.05 (Student’s t test). Data are presented as mean ± SD. (d-f) Mouse BMSCs harvested from *Mapk7^flox/flox^* mice were infected with Ad-GFP or Ad-Cre for 48 h, followed by being induced osteogenic differentiation for 7 days. Ad, adenovirus. (d) Osteogenic differentiation of mouse BMSCs cultured on 24-well plates was determined by alkaline phosphatase staining. (e) Real-time quantitative PCR and (f) western blot analyses in the indicated mouse BMSCs. (n = 3). *P < 0.05 (Student’s t test). Data are presented as mean ± SD.


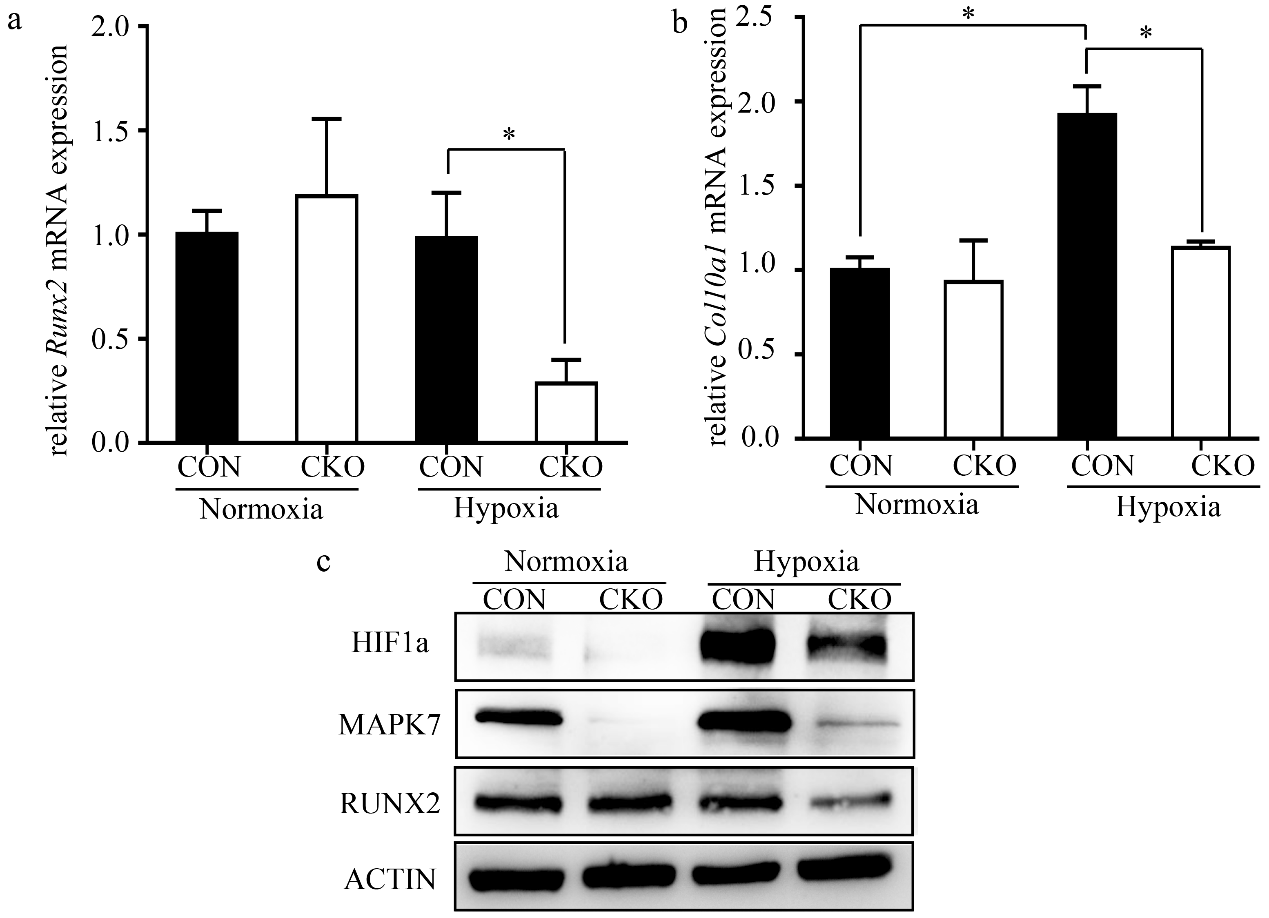


**Fig. S8.** MAPK7 deficiency inhibited the expressions of RUNX2 and COL10A1 under hypoxia. mRNA levels of (a) *Runx2* and (b) *Col10a1* in CON and *Mapk7* CKO chondrocytes cultured under normoxia or hypoxia were measured by real-time quantitative PCR (n = 3). **P* < 0.05 (one-way ANOVA followed by Dunnett’s post hoc test). Data are presented as mean ± SD. (c) Western blot analysis of HIF1α, MAPK7 and RUNX2 in CON and *Mapk7* CKO chondrocytes cultured under normoxic or hypoxic conditions for 48 h.


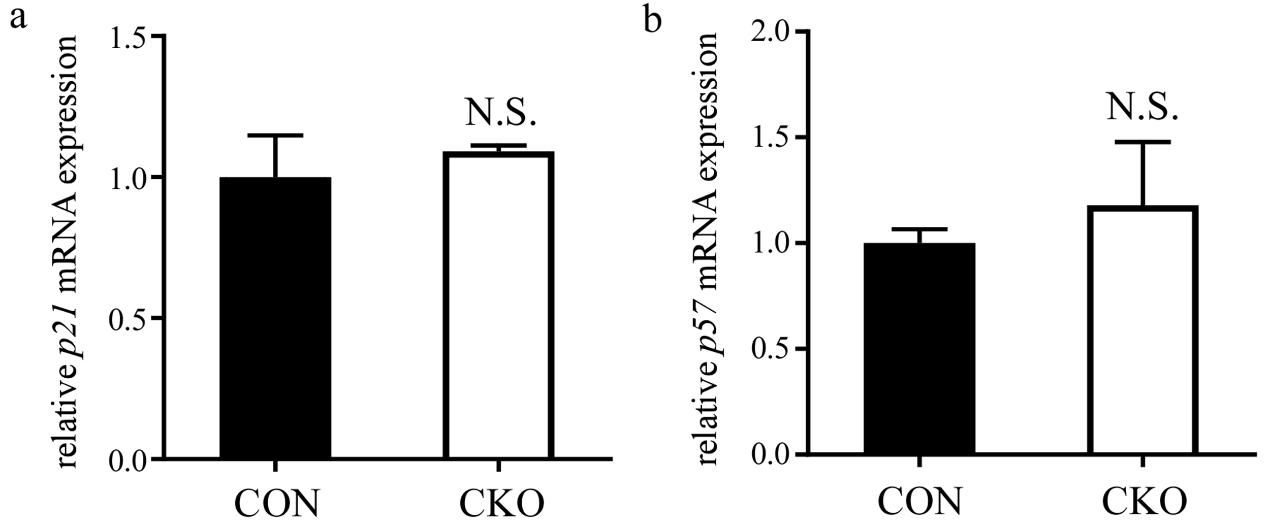


**Fig. S9.** MAPK7 deficiency did not affect the mRNA levels of *p21* and *p57* in growth plate cartilage. Real-time quantitative PCR showed mRNA levels of (a) *p21* and (b) *p57* in growth plate cartilage isolated from CON and *Mapk7* CKO mice at P1 (*n* = 3). **P* < 0.05 (Student’s *t* test). N.S., not significant. Data are presented as mean ± SD.


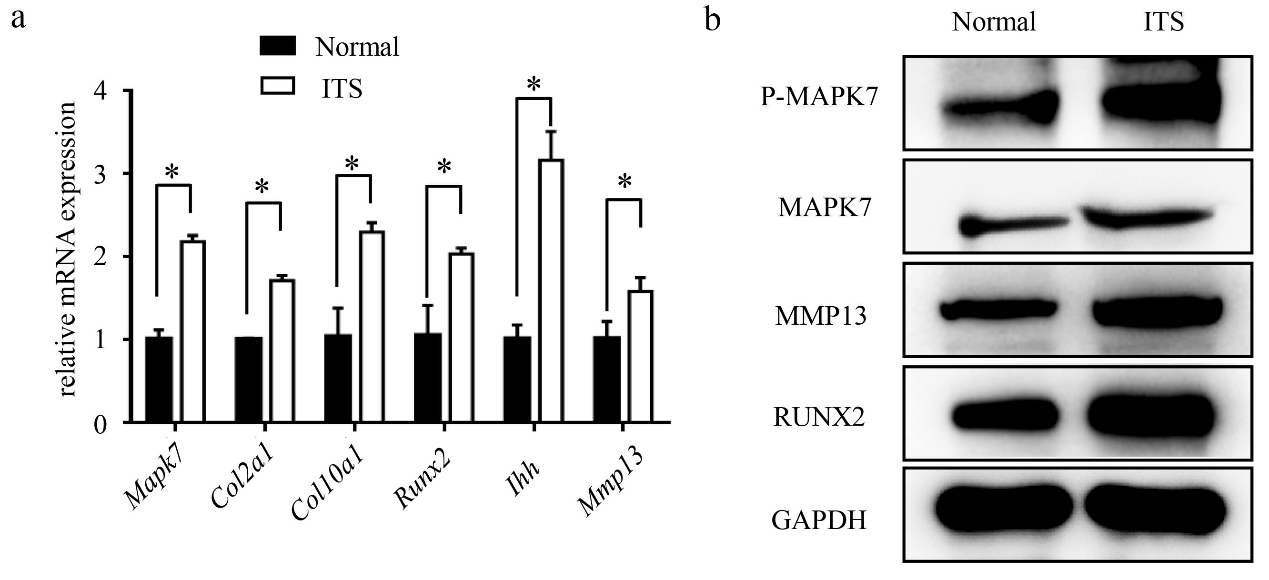


**Fig. S10.** Compared with normal medium, MAPK7 and p-MAPK7 were up-regulated when epiphyseal chondrocytes were cultured in ITS medium. (a) Wild-type chondrocytes were cultured in normal or ITS medium for 7 d, and mRNA levels of *Mapk7*, *Col2a1, Col10a1, Runx2, Ihh,* and *Mmp13* were measured by real-time quantitative PCR (n = 3). **P* < 0.05 (Student’s *t* test). Data are presented as mean ± SD. (b) The expressions of p-MAPK7, MAPK7, MMP13, and RUNX2 in wild-type chondrocytes cultured in normal or ITS medium on day 7 were analyzed by western blot. ITS, insulin-transferrin-selenium medium.

**Supplementary Tables**

**Table S1.** Primer sequences for genotyping.

| Gene | Primer sequence |
| --- | --- |
| Mapk7 loxp | Sense: 5’-CAGGACAGATCCAAATTGTAAGACA -3’ |
|  | Anti-sense: 5’- TAGCCCCAACTTTTTTACAGCTTTC -3’ |
| Col2a1-Cre | Sense: 5’- ATCCGAAAAGAAAACGTTGA -3’ |
|  | Anti-sense: 5’-ATCCAGGTTACGGATATAGT -3’ |

**Table S2.** Primer sequences for real-time quantitative PCR.

| *Gene* | Primer sequence |
| --- | --- |
| *Cyclin B1* | Sense: 5’-GCGTGTGCCTGTGACAGTTA-3’ |
|  | Anti-sense: 5’- CCTAGCGTTTTTGCTTCCCTT-3’ |
| *Gapdh* | Sense: 5’- AGGTCGGTGTGAACGGATTTG-3’ |
|  | Anti-sense: 5’-TGTAGACCATGTAGTTGAGGTCA-3’ |
| *Runx2* | Sense: 5’-CCAACCGAGTCATTTAAGGCT-3’ |
|  | Anti-sense: 5’-GCTCACGTCGCTCATCTTG-3’ |
| *Col10a1* | Sense: 5’-TTCTGCTGCTAATGTTCTTGACC-3’ |
|  | Anti-sense: 5’-GGGATGAAGTATTGTGTCTTGGG-3’ |
| *Sox9* | Sense: 5’- GAGCCGGATCTGAAGAGGGA-3’ |
|  | Anti-sense: 5’- GCTTGACGTGTGGCTTGTTC-3’ |
| *Ihh* | Sense: 5’-CTCTTGCCTACAAGCAGTTCA-3’ |
|  | Anti-sense: 5’-CCGTGTTCTCCTCGTCCTT-3’ |
| *Opn* | Sense: 5’-AGCAAGAAACTCTTCCAAGCAA-3’ |
|  | Anti-sense: 5’-GTGAGATTCGTCAGATTCATCCG-3’ |
| *Mmp13* | Sense: 5’-CTTCTTCTTGTTGAGCTGGACTC -3’ |
|  | Anti-sense: 5’-CTGTGGAGGTCACTGTAGACT-3’ |
| *P57* | Sense: 5’-CGAGGAGCAGGACGAGAATC-3’ |
|  | Anti-sense: 5’-GAAGAAGTCGTTCGCATTGGC-3’ |
| *Col2a1* | Sense: 5’-CTGGTGGAGCAGCAAGAGCAA-3’ |
|  | Anti-sense: 5’-CAGTGGACAGTAGACGGAGGAAAG-3’ |
| *Pcna* | Sense: 5’-TTTGAGGCACGCCTGATCC-3’ |
|  | Anti-sense: 5’-GGAGACGTGAGACGAGTCCAT-3’ |
| *Ki67* | Sense: 5’-ATCATTGACCGCTCCTTTAGGT-3’ |
|  | Anti-sense: 5’-GCTCGCCTTGATGGTTCCT-3’ |
| *Cyclin D1* | Sense: 5’-GCGTACCCTGACACCAATCTC-3’ |
|  | Anti-sense: 5’-CTCCTCTTCGCACTTCTGCTC-3’ |
| *Hif1a* | Sense: 5'-TCAAGTCAGCAACGTGGAAG-3' |
|  | Anti-sense: 5'-TATCGAGGCTGTGTCGACTG-3' |
| *Vegfa* | Sense: 5’-GAGAGAGGCCGAAGTCCTTT-3’ |
|  | Anti-sense: 5’-TTGGAACCGGCATCTTTATC-3′ |
| *Glut1* | Sense: 5’-GGGCATGTGCTTCCAGTATGT-3’ |
|  | Anti-sense: 5’-ACGAGGAGCACCGTGAAGAT-3’ |
| *Pgk1* | Sense: 5’-CTGTGGTACTGAGAGCAGCAAGA-3’ |
|  | Anti-sense: 5’-CAGGACCATTCCAAACAATCTG-3’ |
| *P21* | Sense: 5’-CCTGGTGATGTCCGACCTG-3’ |
|  | Anti-sense: 5’-CCATGAGCGCATCGCAATC-3’ |
| β*-actin* | Sense: 5’-GCACTGTGTTGGCATAGAGG-3’ |
|  | Anti-sense: 5’-GTTCCGATGCCCTGAGGCTCTT-3’ |
| *Alpl* | Sense: 5’-CCAACTCTTTTGTGCCAGAGA-3’ |
|  | Anti-sense: 5’-GGCTACATTGGTGTTGAGCTTTT-3’ |
| *Erk5* | Sense: 5’-TAAACCCTCTAACCTTCTGGTCA-3’ |
|  | Anti-sense: 5’-GAGGTCGATTGCCTGCGTA-3’ |
| *OPN* | Sense: 5’-CTCCATTGACTCGAACGACTC-3’ |
|  | Anti-sense: 5’-CAGGTCTGCGAAACTTCTTAGAT-3’ |
| *OCN* | Sense: 5’-CACTCCTCGCCCTATTGGC-3’ |
|  | Anti-sense: 5’-CCCTCCTGCTTGGACACAAAG-3’ |
| *OSX* | Sense: 5’-TTTGCTCCCCTTAATCCAGCC-3’ |
|  | Anti-sense: 5’-CCTGGCAATTAGGGCAGTCG-3’ |
| *GAPDH* | Sense: 5’-AGAAAAACCTGCCAAATATGATGAC-3’ |
|  | Anti-sense: 5’-TGGGTGTCGCTGTTGAAGTC-3’ |
| *ALPL* | Sense: 5’-ACTGGTACTCAGACAACGAGAT-3’ |
|  | Anti-sense: 5’-ACGTCAATGTCCCTGATGTTATG-3’ |
